# Crystal structures of [Li_7_(*i*-PrO)_3_(C_4_H_10_NO)_3_]_2_O and [Na(*i*-PrOH)_2_(C_8_H_18_NO_2_)]_2_

**DOI:** 10.1107/S2056989020006659

**Published:** 2020-05-29

**Authors:** Rebecca Scheel, Kathrin Louven, Carsten Strohmann

**Affiliations:** a Inorganic Chemistry, TU Dortmund University, Otto-Hahn Str. 6, 44227 Dortmund, Germany

**Keywords:** crystal structure, lithium alkoxide, sodium alkoxide, iso­propanol, amino­alkoxide

## Abstract

The title compounds, hexa­kis­[μ_3_-2-(di­methyl­amino)­ethano­lato]hexa-μ_2_-iso­propano­lato-μ_4_-oxido-tetra­deca­lithium(I), [Li_7_(*i*-PrO)_3_(C_4_H_10_NO)_3_]_2_O (**1**), and {3-[(2-meth­oxy­eth­yl)(meth­yl)amino]-1,1-dimethylpropano­lato}diiso­prop­an­o­lsodium(I), [Na(*i*-PrOH)_2_(C_8_H_18_NO_2_)] (**2**), were crystallized in the presence of 2-propanol (*i*-PrOH, C_3_H_7_OH). The structure **1** has monoclinic symmetry (*C*2/*c*) and the asymmetric unit contains half of the compound. Title compound **2** has triclinic symmetry (*P* ) and the asymmetric unit is half of an inversion-symmetric aggregate. Both compounds consist of an alkali metal, an amino­alkoxide and a 2-propanol compound. Furthermore, the dimeric sodium aggregate **2** is build up by hydrogen bonding through the 2-propanol and the alkoxides. Compound **1** does not exhibit hydrogen bonding, due to the fact that the 2-propanol is deprotonated. In compound **1**, benzene appeared as co-crystallate, but was suppressed by solvent masking because of strong disorder. The formula mass and density do not take account of the solvent.

## Abstract 2

The crystal structures of [Li_7_(*i*-PrO)_3_(C_4_H_10_NO)_3_]_2_O (**1**) and [Na(*i*-PrOH)_2_(C_8_H_18_NO_2_)]_2_ (**2**) were determined at 100 K. In title compound **2**, O—H⋯O hydrogen bonding can be observed which builds up the dimeric structure.

## Chemical context   

The combination of alkali-metal organyls, amino­alkoxides and alcohols is of great inter­est for understanding the behaviour of alkali-metal organyls in deprotonation or metalation reactions. Alkoxides, and especially amino­alkoxides, are used to increase the reactivity of alkali-metal organyls through deaggregation (Lochmann & Janata, 2014 ▸; Caubére, 1993 ▸). As a result of the formation of oligomers, alkali-metal organyls should be deaggregated to get easily accessible metal centers and thus increase the reactivity (Streitwieser *et al.*, 1976 ▸; Gessner *et al.*, 2009 ▸). This deaggregation can be carried out by solvent mol­ecules or Lewis bases, such as amino­alkoxides. Thus the use of amino­alkoxides leads to a highly reactive species, which has great impact on deprotonation or metal­ation reactions with alkali-metal organyls in chemical synthesis (Gros *et al.*, 1995 ▸; Gros *et al.*, 1997 ▸). Previous studies have shown that the reaction of 2-meth­oxy­pyridine with a lithium alkyl and a lithium di­methyl­amino­ethoxide leads to a high yield of the metalated product, while the reaction without the amino­alkoxide only leads to a nucleophilic addition (Gros & Fort, 2002 ▸). Besides 2-meth­oxy­pyridine, the metalation of pyridine and quinoline with a mixture of lithium alkyl and lithium amino­alkoxide can also be observed, which indicates a higher substrate scope and a higher reactivity (Gros & Fort, 2002 ▸). This mixture is a so-called monometallic superbase of the second generation, based on the Lochmann–Schlosser superbase (*n*-butyl­lithium and potassium-*tert*-butoxide) (Schlosser, 1967 ▸; Lochmann *et al.*, 1966 ▸, 1970 ▸, 1972 ▸; Lochmann & Janata, 2014 ▸). In particular, the insertion of the amine function in the alkoxide shows a high stabilization, which is proven by the structure of (LiDMAE)_8_ (Andrews *et al.*, 2002 ▸). The structural behaviour exhibits a high stabilization and a broader approach to deprotonation possibilities. Therefore, the use of an alkali-metal amino­alkoxide in combination with an alkali-metal organyl is a potential monometallic superbase of the second generation. But, because of the synthesis of the lithium amino­alkoxide with the amino­alcohol (di­methyl­amino­ethanol), which is added *in situ* to the butyl­lithium solution, reaction mixtures can still contain the pure amino­alcohol (Gros *et al.*, 1997 ▸). In general, alcohols also affect the structural and chemical behaviour of alkali-metal organyls. As well as the synthesis of the amino­alkoxides, they are also used during the synthesis of alkali-metal organyls such as *tert*-butyl­lithium. To gain higher yields, it is reported that the addition of small amounts of *tert*-butyl alcohol improves the yield to 80% instead of 40% (Smith, 1974 ▸). Consequently, in metalation and deprotonation reactions containing amino­alkoxides and alkali-metal organyls, small amounts of alcohols can also be available. Usually the alcohol, which is added during the synthesis of the organometal reagents, is metalated. By adding a second alcohol, the amino­alcohol, it is not clear whether the previous alcohol is still metalated. Therefore, the influence of these amounts of alcohol is of great inter­est for understanding the reactivity and the mechanistic behaviour. The structures obtained here reflect the inter­action of the reagents and can provide insights into the influence of alcohols, which occur during the synthesis of the alkoxide or the synthesis of the organometal reagent. In particular, the insertion of an alcohol that is not based on the amino­alkoxide generates high inter­est, because it can be concluded whether the amino­alcohol is deprotonated to form the alkali-metal alkoxide or the additional alcohol is deprotonated. The combination of alkali-metal organyls, amino­alkoxides and alcohols is therefore of great inter­est for understanding the synergistic effect.
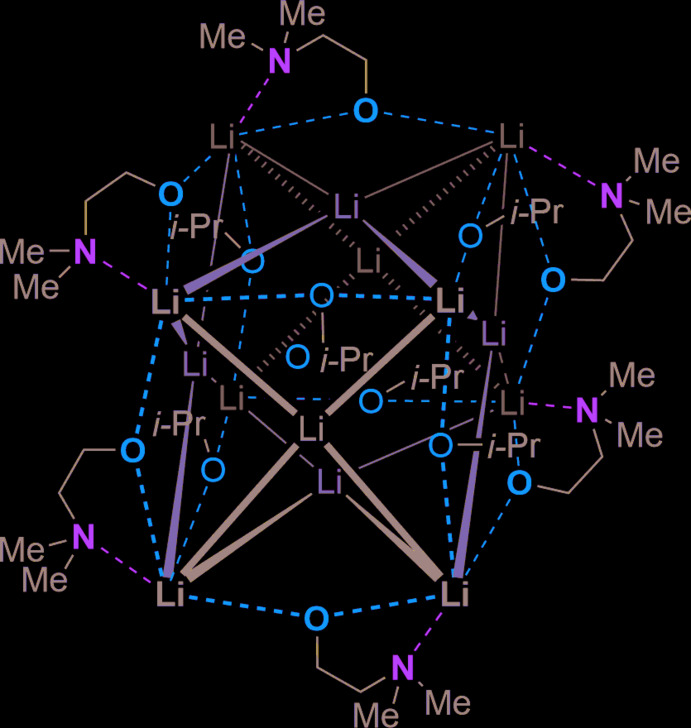
 
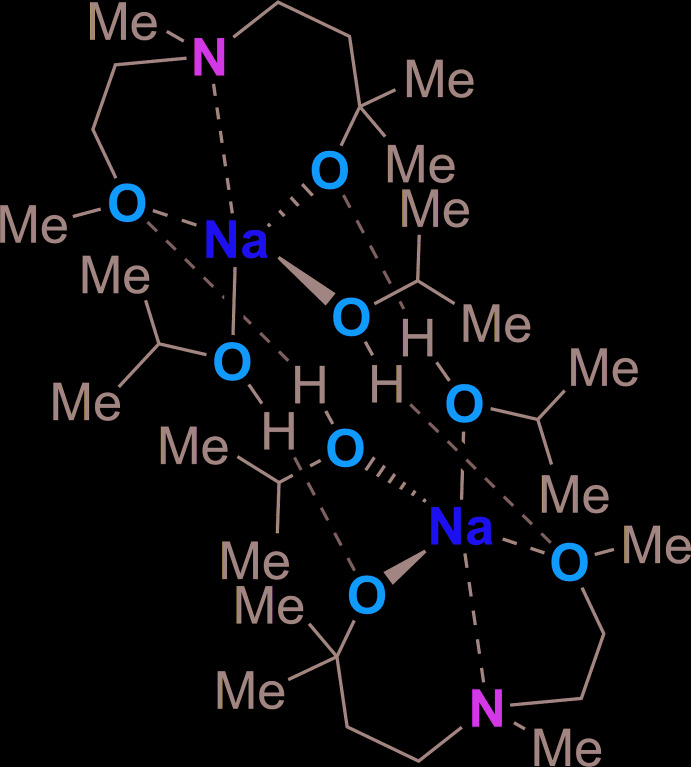


## Structural commentary   

**Figure 1:**
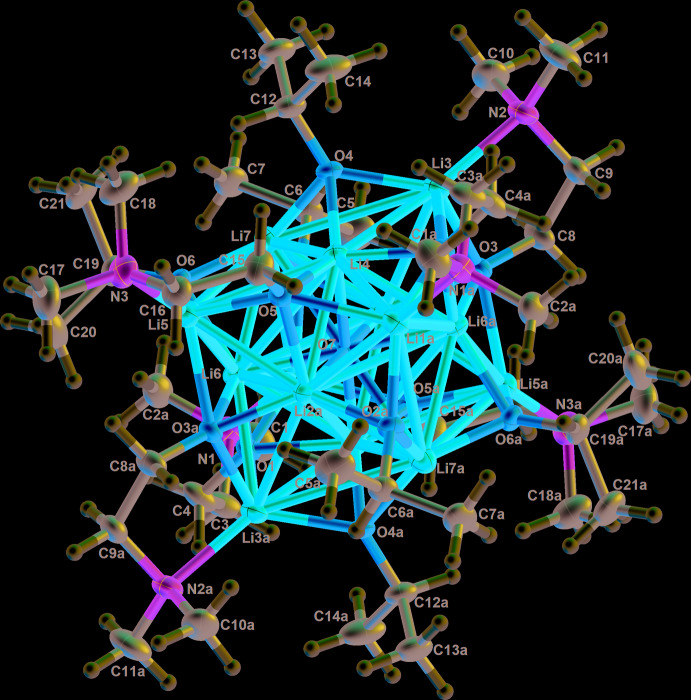
Mol­ecular structure of compound **1** [Li_7_(*i*-PrO)_3_(C_4_H_10_NO)_3_]_2_O with atom labeling. The asymmetric unit is half of the compound, symmetry operation a = 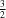 − *x*, 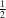 − *y*, 1 − *z*.

The title compounds **1** and **2** crystallize at 193 K in the presence of iso­propanol. Both structures contain an alkali metal, an amino­alkoxide and iso­propanol. Compound **1** crystallizes in the monoclinic space group *C*2/*c* and the asymmetric unit contains half of the compound, which is built up by a twofold rotation axis. Compound **1** consists of lithium triangles, which are capped by the oxygen atom of the isopropoxide and the amino­alkoxide. Di­methyl­amino­ethanoxide, whose nitro­gen atom also coordinates a lithium center, is used as an alkoxide. The mol­ecule contains fourteen lithium centers, which are arranged as a distorted face-centered cube around an oxygen center. The oxygen center is located on a special position (4*c*, 0.75, 0.25, 0.5) and is dianionic. The structure is shown in Fig. 1 ▸ and selected bond lengths and angles are given in Table 1 ▸. The distances between the oxygen atom of the amino­alkoxide and the next lithium center are 1.901 (2) Å for Li2—O3, 1.893 (2) Å for Li6—O1 and 1.899 (2) Å for Li4—O5. Comparing these to a similar structure in the literature, containing the same amino­alkoxide, the bond length is, at 1.899 (2) Å, nearly in the same range (Andrews *et al.*, 2002 ▸). Furthermore, the bond lengths of the oxygen atom and the lithium center, which is coordinated by a nitro­gen atom, are slightly elongated with 1.989 (2) Å (Li3—O3), 1.989 (2) Å (Li1—O1) and 1.997 (2) Å (O5—Li5) because of the rigid structure of the amino­alkoxide and the formed inter­nal ring. The bond lengths between the nitro­gen atom of the amino­alkoxide and the lithium center vary between 2.141 (3) Å for Li3—N2, 2.157 (2) Å for Li1—N1 and 2.125 (2) Å for Li5—N3, which is slightly shorter than the bond length in the literature for the (LiDMAE)_8_ compound [2.189 (2) Å; Andrews *et al.*, 2002 ▸]. Moreover, the bond lengths of the oxygen atom of the isopropoxide and the lithium centers amount to 1.920 (2) Å for Li2—O2, 1.918 (2) Å for Li4—O4 and 1.910 (2) Å for Li6—O6, respectively. Thus, they are slightly shorter compared to bonds in the literature, which vary between 1.953 (8) and 1.962 (7) Å (Crozier *et al.*, 2013 ▸). The bond length of the inter­nal oxygen atom O7 exhibits a very long distance to the opposite lithium center Li7, with a bond length of 2.554 (3) Å. The distances to the other lithium centers Li2, Li4 and Li6 are shorter and come to bond lengths of 2.003 (2) Å for O7—Li2, 2.007 (2) Å for O7—Li4, and 1.997 (2) Å for O7—Li6. The bond angles of the lithium centers and the oxygen of the amino­alkoxide, Li2—O3—Li3, Li1—O1—Li6 and Li4—O5—Li5, are 93.81 (10), 94.45 (10) and 93.77 (10)°, respectively, and therefore wider than the bond angles in comparable structures [80.55 (9)°; Andrews *et al.*, 2002 ▸]. Moreover, the bond angles of the nitro­gen atom of the amino­alkoxide, the lithium center and the oxygen atom of the amino­alkoxide are 88.05 (10)° for N2—Li3—O3, 88.06 (10)° for N1—Li1—O1 and 88.55 (10)° for N3—Li5—O5. Compared to a structure in the literature with an angle of 90.25 (10)° (Andrews *et al.*, 2002 ▸), the angles in the observed compound are compressed. The bond angle of the lithium atoms and the oxygen atom of the isopropoxide are 79.26 (10)° (Li1—O2—Li2), 79.25 (10)° (Li3—O4—Li4) and 79.35 (10)° (Li5—O6—Li6). In contrast, the angles of the oxygen atom of the isopropoxide and the outermost lithium center Li7 are much wider at 116.92 (11)° for Li1—O2—Li7, 116.33 (11)° for Li7—O4—Li3 and 116.55 (12)° for Li5—O6—Li7.

**Figure 2:**
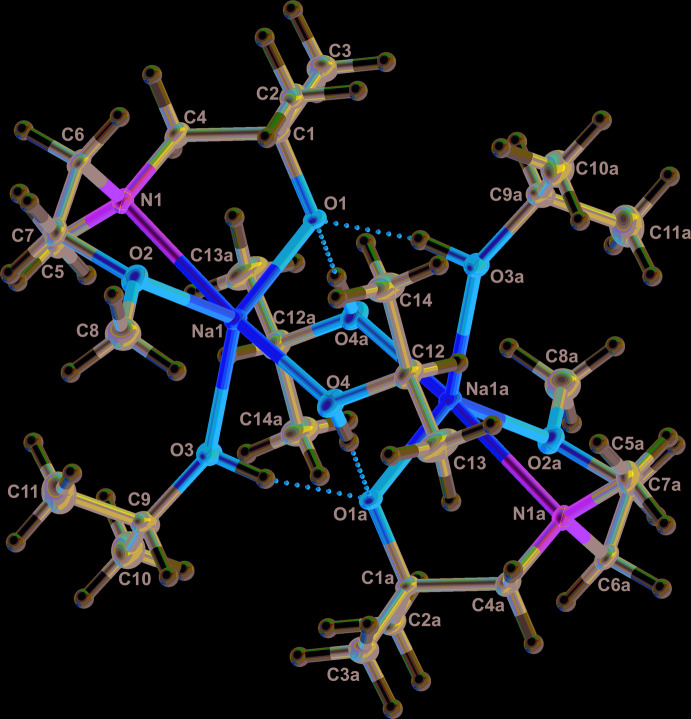
Mol­ecular structure of compound **2** [Na(*i*-PrOH)_2_(C_8_H_18_NO_2_)]_2_ with atom labeling. The asymmetric unit is half of the compound, symmetry operation a = 1 − *x*, 1 − *y*, 1 − *z*.

Compound **2** crystallizes in the triclinic space group *P* 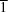 and the asymmetric unit contains half of the mol­ecule. [Na(*i*-PrOH)_2_(C_8_H_18_NO_2_)]_2_ is a sodium dimer with a deprotonated alcohol that coordinates the sodium atom with its three donor atoms. The deprotonated alcohol *N*-meth­yl(2-meth­oxy­ethyl­amino)-2-methyl-2-propanol consists of a central nitro­gen atom and two oxygen atoms. Therefore, it has excellent properties as a donating ligand. The structure of the compound is given in Fig. 2 ▸ and selected bond lengths and angles are given in Table 2 ▸. The asymmetric unit contains a central sodium atom with a trigonal–bipyramidal coordination sphere. The nitro­gen atom of the amino­alkoxide builds up the top pyramid site, while one iso­propanol builds up the opposite pyramid site. The two oxygen atoms of the amino­alkoxide and another iso­propanol are located triangularly around the sodium atom. The distance between the sodium atom Na1 and the nitro­gen atom N1 is 2.5707 (11) Å, which is an elongated bond length in comparison to a literature structure [2.528 (2) Å; Marszałek-Harych *et al.*, 2020 ▸]. The bond lengths between the sodium atom and the oxygen atom of the amino­alkoxide are 2.3736 (10) Å for Na1—O2 and 2.2970 (10) Å for Na1—O1, which is in accordance with a similar compound in the literature with bond lengths of 2.239 (2) and 2.352 (2) Å (Marszałek-Harych *et al.*, 2020 ▸). Moreover, the bond lengths between the sodium atom and the oxygen atoms of the iso­propanols are 2.3905 (10) Å (Na1—O4) and 2.2998 (10) Å (Na1—O3). A similar compound in the literature exhibits an Na—O bond length of 2.402 (6) Å and is therefore much longer (Edema *et al.*, 1991 ▸). The bond angles N1—Na1—O1 and N1—Na1—O2 are 73.22 (3) and 69.39 (3)°, respectively. Compared to a bond angle in the literature of 66.7 (4)° (Schüler *et al.*, 2019 ▸), the angles in the title structure are much wider. That might be traced back to the fact that the angles are limited because of the rigid structure of the amino­alkoxide. As the coordination sphere of the sodium atom is arranged like a trigonal bipyramid, the bond angles correspond to this. One iso­propanol mol­ecule is arranged in the triangular sphere around the sodium and therefore exhib­its an angle of 109.84 (4)° for N1—Na1—O3. The other iso­propanol is arranged in the opposite pyramid site of the amine function and shows an angle of 160.83 (4)° for N1—Na1—O4, respectively.

**Figure 3:**
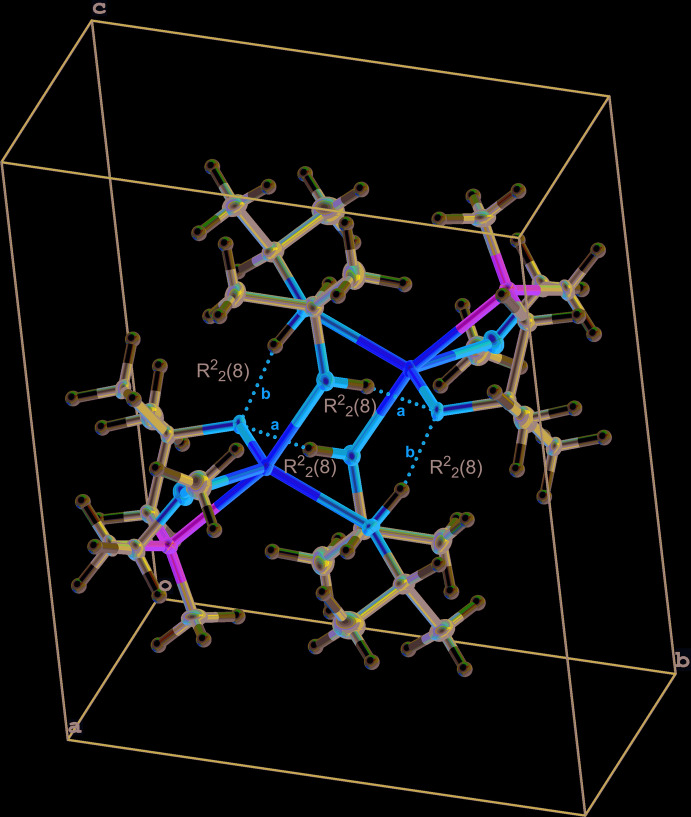
View of the unit cell of **2**, Hydrogen bonding is shown and the graph-set motifs are labelled (*Mercury;* Macrae *et al.*, 2008 ▸). Symmetry operation a = 1 − *x*, 1 − *y*, 1 − *z*.

**Figure 4:**
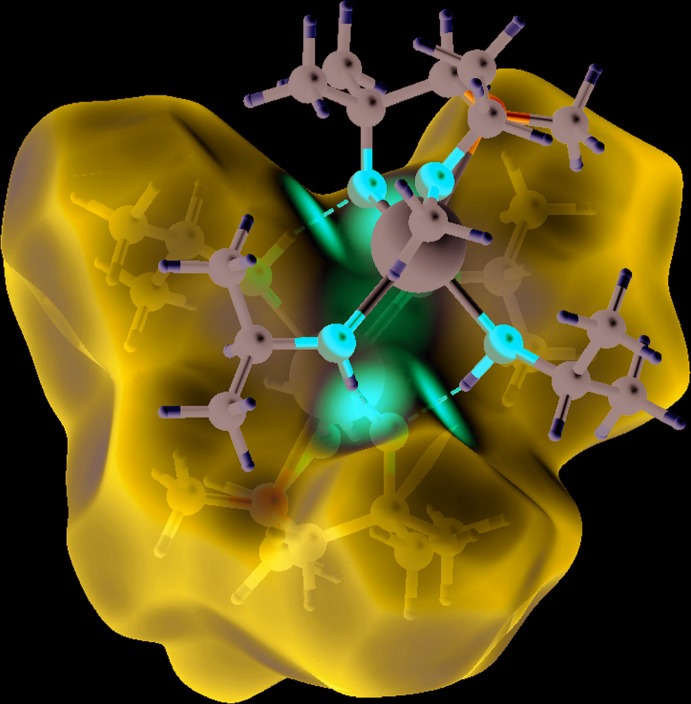
Hirshfeld surface analysis (*CrystalExplorer17;* Turner *et al.*, 2017 ▸) of compound **2** illustrating the hydrogen bonding. Symmetry operation a = 1 − *x*, 1 − *y*, 1 − *z*.

In this dimeric structure, the two sodium centers are bridged by iso­propanol mol­ecules. From the hydrogen atoms of the iso­propanol, a hydrogen bond to the oxygen atoms of the amino­alkoxide is formed. Details of the hydrogen bonding are given in Table 3 ▸. As hydrogen bonds are present in the compound, it can be shown that the amino­alkoxide oxygen is more acidic than the alcohol function of the iso­propanol. The graph-set motifs of the hydrogen bonds *a* and *b* are 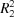
(8) and 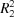
(8), respectively, and are shown in Fig. 3 ▸ (*Mercury;* Macrae *et al.*, 2020 ▸). In addition, a Hirshfeld surface analysis has been carried out with a *d*_norm_ property over a range of −0.7978 to +1.3992 a.u. The characteristic red spots in Fig. 4 ▸ (*CrystalExplorer17;* Turner *et al.*, 2017 ▸) indicate inter­nal hydrogen bonding.

In **1** as well as in **2**, the alcohol characterizes the structural motif. In **1**, the iso­propanol is crucial for the saturation of the coordination sphere of the lithium atoms. In addition, it serves not only as the anionic part, but also as a ligand. In **2**, the iso­propanol is located opposite the nitro­gen of the amino­alkoxide. It serves as a ligand that bridges the dimeric structure by hydrogen bonding. Therefore, alcohols affect the structural motifs and thus inter­act with the reagents.

## Supra­molecular features   

**Figure 5:**
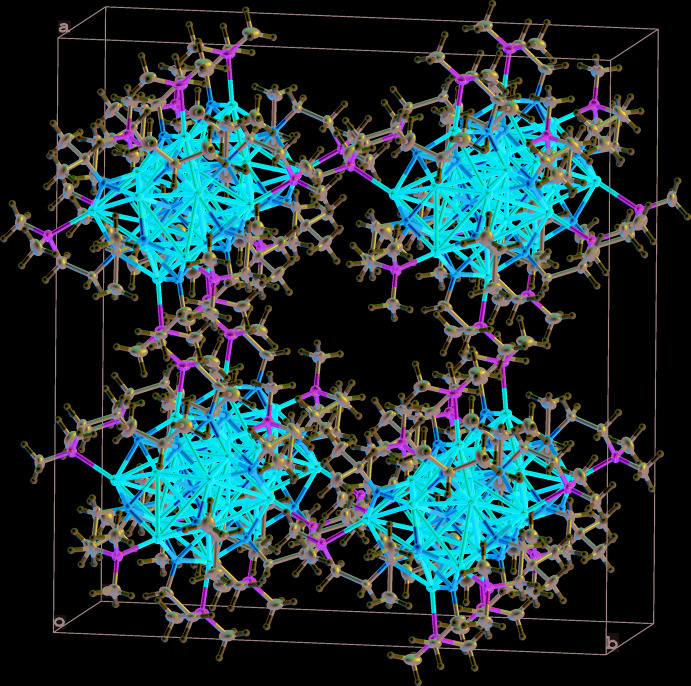
View of the crystal packing of **1** parallel to the *ac* plane and the *bc* plane.

The title compound [Li_7_(*i*-PrO)_3_(C_4_H_10_NO)_3_]_2_O (**1**) is a dimeric mol­ecule where the asymmetric unit is half of the mol­ecule and the full structure is build up by a twofold rotation axis. It is packed parallel to the *ac* plane and to the *bc* plane, as shown in Fig. 5 ▸. The second title compound, [Na(*i*-PrOH)_2_(C_8_H_18_NO_2_)]_2_ (**2**), is also a dimeric mol­ecule, the asymmetric unit being half of an inversion symmetric aggregate. The mol­ecules are packed parallel to the *ab* plane and the *bc* plane.

## Database survey   

Other examples of crystallographically characterized complexes containing an alkali metal center and a directly to the metal center coordinated iso­propanol are C_40_H_88_Cr_2_Na_4_O_12_ (Edema *et al.*, 1991 ▸) and C_26_H_66_CeLiN_2_O_4_Si_4_ (Crozier *et al.*, 2013 ▸). Both compounds, **1** and **2**, contain an amino­alkoxide as well as iso­propanol. Examples of crystallographically characterized complexes containing a lithium amino­alkoxide are C_54_H_36_Li_6_N_6_O_6_ (Begley & Rajeswaran, 2006 ▸), C_20_H_43_LiN_2_O_2_Si (Bauer & Strohmann, 2014 ▸), C_36_H_72_Li_6_N_6_O_6_·0.5 C_4_H_10_ (Strohmann *et al.*, 2004 ▸), C_42_H_56_Li_2_N_2_O_2_Si_2_ (Unkelbach *et al.*, 2012 ▸), C_13_H_31_LiN_2_O_2_Si (Colquhoun & Strohmann, 2012 ▸) and C_18_H_36_Li_2_N_2_O_4_ (Kroesen *et al.*, 2017 ▸). A very rare example is C_32_H_80_Li_8_N_8_O_8_ (Andrews *et al.*, 2002 ▸), because the crystallographically characterized complex contains the same amino­alkoxide used in **1**. Furthermore, crystallographically characterized sodium complexes containing an amino­alkoxide are C_16_H_34_N_2_NaO_6_I (Fronczek *et al.*, 1983 ▸), C_16_H_44_N_3_NaO_2_Si_2_ (Schüler *et al.*, 2019 ▸), C_18_H_48_N_3_NaO_3_Si_4_ (Thalangamaarachchige *et al.*, 2017 ▸), C_56_H_84_MgNa_2_N_4_O_4_ (Hevia *et al.*, 2006 ▸) and C_35_H_58_N_4_Na_2_O_4_ (Hevia *et al.*, 2006 ▸). In addition, a crystallographically characterized complex containing a penta­coordinated sodium atom and amine and oxygen functions as ligands are C_50_H_88_N_2_Na_2_O_6_, C_74_H_104_N_2_Na_2_O_6_ and C_48_H_80_N_2_Na_2_O_8_ (Marszałek-Harych *et al.*, 2020 ▸).

## Synthesis and crystallization   

**Compound 1 [Li_7_(** ***i*** **-PrO)_3_(C_4_H_10_NO)_3_]_2_O:**

Di­methyl­amino ethanol (100 mg, 1.12 mmol, 1.0 eq.) was added to 1.0 mL of diethyl ether and cooled to 195 K. After that, *n*-butyl­lithium in hexane (*c* = 2.5 mol L ^– 1^, 0.9 mL, 2.24 mmol, 2.0 eq.) was added dropwise and 0.5 mL of benzene were added for crystallization. The reaction mixture was left to stand for 15 min, while warming up to 218 K. Then it was stored at 193 K in the coolant iso­propanol. Colorless crystals were formed after 45 d. During storage, the coolant iso­propanol seems to have diffused into the vessel, leading to the incorporation of iso­propanol in the crystal structure of compound **1**.

**Compound 2 [Na(** ***i*** **-PrOH)_2_(C_8_H_18_NO_2_)]_2_:**

Sodium-*N*-meth­yl(2-meth­oxy­ethyl­amino)-2-methyl-2-prop­anoxide (49.5 mg, 0.27 mmol, 1.0 eq.) was dissolved in 2.0 mL of diethyl ether and cooled to 195 K. Then *n*-butyl­lithium in hexane (*c* = 2.5 mol L ^– 1^, 0.13 mL, 0.33 mmol, 1.2 eq.) was added and the reaction mixture was left to stand in the cooling bath for 15min. The reaction mixture warmed up to 218 K and was then stored at 193 K in the coolant iso­propanol. Colorless crystals were formed after 60 d. During the storage, the coolant iso­propanol seems to have diffused into the vessel, leading to the incorporation of iso­propanol in the crystal structure of compound **2**.

## Refinement   

Crystal data, data collection and structure refinement details are summarized in Table 4 ▸. All of the hydrogen atoms were placed in geometrically calculated positions and were each assigned a fixed isotropic displacement parameter based on a riding-model: C—H = 0.98–1.0 Å with *U*_iso_(H) = 1.5*U*_eq_(C-meth­yl) and 1.2*U*_eq_(C) for other H atoms. Apart from this, the O-bound hydrogen atoms of compound **2** were located in the difference-Fourier maps and refined independently. In compound **1**, benzene appeared as co-crystallate, but was suppressed by solvent masking because of strong disorder.

## Supplementary Material

Crystal structure: contains datablock(s) 1, 2. DOI: 10.1107/S2056989020006659/vm2233sup1.cif

Structure factors: contains datablock(s) 1. DOI: 10.1107/S2056989020006659/vm22331sup2.hkl

Structure factors: contains datablock(s) 2. DOI: 10.1107/S2056989020006659/vm22332sup3.hkl

CCDC references: 2004433, 2004432

Additional supporting information:  crystallographic information; 3D view; checkCIF report

## Figures and Tables

**Table 1:** Selected geometric parameters (Å, °) for **1**

Li2—O3	1.901 (2)	Li5—N3	2.125 (2)
Li6—O1	1.893 (2)	Li2—O2	1.920 (2)
Li4—O5	1.899 (2)	Li4—O4	1.918 (2)
Li3—O3	1.989 (2)	Li6—O6	1.910 (2)
Li1—O1	1.989 (2)	Li7—O7	2.554 (3)
Li5—O5	1.997 (2)	Li2—O7	2.003 (2)
Li3—N2	2.141 (3)	Li4—O7	2.007 (2)
Li1—N1	2.157 (2)	Li6—O7	1.997 (2)
			
Li2—O3—Li3	93.81 (10)	Li1—O2—Li2	79.26 (10)
Li1—O1—Li6	94.45 (10)	Li3—O4—Li4	79.25 (10)
Li4—O5—Li5	93.77 (10)	Li5—O6—Li6	79.35 (10)
N1—Li1—O1	88.06 (10)	Li7—O4—Li3	116.33 (11)
N3—Li5—O5	88.55 (10)	Li1—O2—Li7	116.92 (11)
N2—Li3—O3	88.05 (10)	Li5—O6—Li7	116.55 (11)

**Table 2:** Selected geometric parameters (Å, °) for **2**

Na1—O2	2.3736 (10)	Na1—O4	2.3905 (10)
Na1—O1	2.2970 (10)	Na1—O3	2.2998 (10)
Na1—N1	2.5707 (11)		
			
N1—Na1—O1	73.22 (3)	N1—Na1—O2	69.39 (3)
N1—Na1—O3	109.84 (4)	N1—Na1—O4	160.83 (4)

**Table 3:** Hydrogen-bond geometry (Å, °) for **2** [Chem scheme1]

*D*—H⋯*A*	*D*—H	H⋯*A*	*D*⋯*A*	*D*—H⋯*A*
O3—H3⋯O1^i^	0.92 (2)	1.65 (2)	2.5442 (12)	165 (2)
O4—H4⋯O1^i^	0.86 (2)	1.75 (2)	2.5894 (12)	164.4 (19)

Symmetry code: (i) 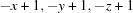.

**Table 4:** Experimental details

	**1**	**2**
Crystal data		
Chemical formula	[Li_14_(C_3_H_7_O)_6_(C_4_H_10_NO)_6_O]	[Na(C_3_H_8_O)_2_(C_8_H_18_NO_2_)]
*M*_r_	996.45	303.41
Crystal system, space group	Monoclinic, *C*2/*c*	Triclinic, *P* 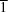
Temperature (K)	100	100
*a*, *b*, *c* (Å)	25.0880 (18), 23.1071 (19), 12.5875 (11)	9.9339 (6), 10.3002 (7), 11.0874 (7)
α, β, γ (°)	90, 95.806 (3), 90	103.333 (2), 108.132 (2), 111.845 (2)
*V* (Å^3^)	7259.7 (10)	920.33 (10)
*Z*	4	2
Radiation type	Mo *K*α	Mo *K*α
μ (mm^−1^)	0.06	0.10
Crystal size (mm)	0.26 × 0.14 × 0.1	0.29 × 0.27 × 0.19

Data collection		
Diffractometer	Bruker D8 VENTURE area detector	Bruker D8 VENTURE area detector
Absorption correction	Multi-scan (*SADABS*; Bruker, 2016 ▸)	Multi-scan (*SADABS*; Bruker, 2016 ▸)
*T*_min_, *T*_max_	0.715, 0.746	0.703, 0.746
No. of measured, independent and observed [*I* > 2σ(*I*)] reflections	121411, 7914, 6608	21708, 4399, 3558
*R*_int_	0.045	0.040
(sin θ/λ)_max_ (Å^−1^)	0.639	0.659

Refinement		
*R*[*F* ^2^ > 2σ(*F* ^2^)], *wR*(*F* ^2^), *S*	0.050, 0.159, 1.06	0.039, 0.095, 1.03
No. of reflections	7914	4399
No. of parameters	462	197
H-atom treatment	H-atom parameters constrained	H atoms treated by a mixture of independent and constrained refinement
Δρ_max_, Δρ_min_ (e Å^−3^)	0.27, −0.28	0.31, −0.27

Computer programs: *APEX2* and *SAINT* (Bruker, 2016 ▸), *SHELXT2014/5* (Sheldrick, 2015*a* ▸), *SHELXL2014/7* (Sheldrick, 2015*b* ▸) and *OLEX2* (Dolomanov *et al.*, 2009 ▸).

## supplementary crystallographic information

### Hexakis[µ3-2-(dimethylamino)ethanolato]hexa-µ2-isopropanolato-µ4-oxido-tetradecalithium(I) (1) Crystal data

**Table:**

[Li_14_(C_3_H_7_O)_6_(C_4_H_10_NO)_6_O]	*F*(000) = 2168
*M**_r_* = 996.45	*D*_x_ = 0.912 Mg m^−^^3^
Monoclinic, *C*2/*c*	Mo *K*α radiation, λ = 0.71073 Å
*a* = 25.0880 (18) Å	Cell parameters from 9461 reflections
*b* = 23.1071 (19) Å	θ = 2.4–29.4°
*c* = 12.5875 (11) Å	µ = 0.06 mm^−^^1^
β = 95.806 (3)°	*T* = 100 K
*V* = 7259.7 (10) Å^3^	Block, colourless
*Z* = 4	0.26 × 0.14 × 0.1 mm

### Hexakis[µ3-2-(dimethylamino)ethanolato]hexa-µ2-isopropanolato-µ4-oxido-tetradecalithium(I) (1) Data collection

**Table:**

Bruker D8 VENTURE area detector diffractometer	7914 independent reflections
Radiation source: microfocus sealed X-ray tube, Incoatec Iµs	6608 reflections with *I* > 2σ(*I*)
HELIOS mirror optics monochromator	*R*_int_ = 0.045
Detector resolution: 10.4167 pixels mm^-1^	θ_max_ = 27.0°, θ_min_ = 2.4°
ω and φ scans	*h* = −31→32
Absorption correction: multi-scan (SADABS; Bruker, 2016)	*k* = −29→29
*T*_min_ = 0.715, *T*_max_ = 0.746	*l* = −16→16
121411 measured reflections	

### Hexakis[µ3-2-(dimethylamino)ethanolato]hexa-µ2-isopropanolato-µ4-oxido-tetradecalithium(I) (1) Refinement

**Table:**

Refinement on *F*^2^	Primary atom site location: dual
Least-squares matrix: full	Hydrogen site location: inferred from neighbouring sites
*R*[*F*^2^ > 2σ(*F*^2^)] = 0.050	H-atom parameters constrained
*wR*(*F*^2^) = 0.159	*w* = 1/[σ^2^(*F*_o_^2^) + (0.0974*P*)^2^ + 4.4307*P*] where *P* = (*F*_o_^2^ + 2*F*_c_^2^)/3
*S* = 1.06	(Δ/σ)_max_ = 0.001
7914 reflections	Δρ_max_ = 0.27 e Å^−^^3^
462 parameters	Δρ_min_ = −0.28 e Å^−^^3^
0 restraints	

### Hexakis[µ3-2-(dimethylamino)ethanolato]hexa-µ2-isopropanolato-µ4-oxido-tetradecalithium(I) (1) Special details

**Table:**

Geometry. All esds (except the esd in the dihedral angle between two l.s. planes) are estimated using the full covariance matrix. The cell esds are taken into account individually in the estimation of esds in distances, angles and torsion angles; correlations between esds in cell parameters are only used when they are defined by crystal symmetry. An approximate (isotropic) treatment of cell esds is used for estimating esds involving l.s. planes.

### Hexakis[µ3-2-(dimethylamino)ethanolato]hexa-µ2-isopropanolato-µ4-oxido-tetradecalithium(I) (1) Fractional atomic coordinates and isotropic or equivalent isotropic displacement parameters (Å^2^)

**Table:**

	*x*	*y*	*z*	*U*_iso_*/*U*_eq_	Occ. (<1)
O1	0.82870 (3)	0.34901 (4)	0.50492 (7)	0.0183 (2)	
O6	0.79419 (4)	0.27930 (4)	0.73760 (7)	0.0202 (2)	
O5	0.77811 (3)	0.14434 (4)	0.63558 (7)	0.0186 (2)	
O4	0.66955 (4)	0.23713 (4)	0.67690 (7)	0.0210 (2)	
O3	0.63408 (3)	0.27145 (4)	0.41861 (7)	0.0192 (2)	
O2	0.70891 (4)	0.36513 (4)	0.60060 (7)	0.0208 (2)	
O7	0.7500	0.2500	0.5000	0.0109 (2)	
N1	0.80465 (5)	0.47063 (5)	0.51979 (10)	0.0279 (3)	
N3	0.84936 (5)	0.14280 (5)	0.82980 (9)	0.0249 (3)	
N2	0.54215 (5)	0.23333 (6)	0.51819 (11)	0.0323 (3)	
C1	0.8202 (3)	0.4929 (3)	0.4105 (6)	0.0379 (17)	0.239 (3)
H1A	0.8529	0.5163	0.4219	0.057*	0.239 (3)
H1B	0.7911	0.5165	0.3759	0.057*	0.239 (3)
H1C	0.8266	0.4598	0.3647	0.057*	0.239 (3)
C1A	0.78645 (10)	0.52463 (9)	0.4698 (2)	0.0470 (7)	0.761 (3)
H1AA	0.8148	0.5538	0.4815	0.071*	0.761 (3)
H1AB	0.7545	0.5381	0.5012	0.071*	0.761 (3)
H1AC	0.7777	0.5185	0.3930	0.071*	0.761 (3)
C2	0.7823 (3)	0.5188 (3)	0.5687 (8)	0.051 (2)	0.239 (3)
H2A	0.8088	0.5501	0.5770	0.077*	0.239 (3)
H2B	0.7722	0.5076	0.6389	0.077*	0.239 (3)
H2C	0.7505	0.5322	0.5238	0.077*	0.239 (3)
C2A	0.81827 (10)	0.48120 (10)	0.63667 (19)	0.0438 (6)	0.761 (3)
H2AA	0.8261	0.4442	0.6731	0.066*	0.761 (3)
H2AB	0.7879	0.4998	0.6660	0.066*	0.761 (3)
H2AC	0.8497	0.5064	0.6474	0.066*	0.761 (3)
C3	0.8508 (3)	0.4442 (3)	0.5765 (6)	0.0324 (16)	0.239 (3)
H3A	0.8422	0.4326	0.6485	0.039*	0.239 (3)
H3B	0.8804	0.4727	0.5853	0.039*	0.239 (3)
C3A	0.85140 (8)	0.44932 (8)	0.47343 (17)	0.0296 (5)	0.761 (3)
H3AA	0.8811	0.4773	0.4884	0.035*	0.761 (3)
H3AB	0.8432	0.4468	0.3950	0.035*	0.761 (3)
C4	0.86954 (5)	0.38992 (6)	0.51670 (13)	0.0292 (3)	
H4AA	0.8999	0.3764	0.4786	0.035*	0.761 (3)
H4AB	0.8825	0.3936	0.5934	0.035*	0.761 (3)
H4BC	0.8795	0.4014	0.4455	0.035*	0.239 (3)
H4BD	0.9016	0.3729	0.5575	0.035*	0.239 (3)
C5	0.87176 (12)	0.32028 (16)	0.8393 (3)	0.0382 (8)	0.830 (12)
H5A	0.8826	0.2830	0.8716	0.057*	0.830 (12)
H5B	0.8828	0.3516	0.8893	0.057*	0.830 (12)
H5C	0.8889	0.3255	0.7733	0.057*	0.830 (12)
C5A	0.8626 (12)	0.3440 (17)	0.8112 (15)	0.078 (8)	0.170 (12)
H5AA	0.8914	0.3254	0.7767	0.116*	0.170 (12)
H5AB	0.8775	0.3615	0.8788	0.116*	0.170 (12)
H5AC	0.8459	0.3741	0.7642	0.116*	0.170 (12)
C6	0.81072 (15)	0.32148 (10)	0.8136 (2)	0.0212 (6)	0.830 (12)
H6	0.8002	0.3603	0.7834	0.025*	0.830 (12)
C6A	0.8264 (8)	0.3052 (15)	0.8302 (15)	0.056 (6)	0.170 (12)
H6A	0.8487	0.2719	0.8596	0.067*	0.170 (12)
C7	0.78459 (7)	0.31309 (7)	0.91547 (11)	0.0353 (4)	
H7AA	0.7456	0.3139	0.8994	0.053*	0.830 (12)
H7AB	0.7958	0.3442	0.9657	0.053*	0.830 (12)
H7AC	0.7955	0.2757	0.9473	0.053*	0.830 (12)
H7BD	0.7577	0.3418	0.8894	0.053*	0.170 (12)
H7BE	0.8033	0.3263	0.9832	0.053*	0.170 (12)
H7BF	0.7670	0.2760	0.9264	0.053*	0.170 (12)
C8	0.78634 (6)	0.09334 (6)	0.69403 (12)	0.0303 (3)	
H8AA	0.7838	0.0600	0.6444	0.036*	0.876 (3)
H8AB	0.7577	0.0892	0.7422	0.036*	0.876 (3)
H8BC	0.8171	0.0720	0.6700	0.036*	0.124 (3)
H8BD	0.7542	0.0684	0.6824	0.036*	0.124 (3)
C9	0.7976 (5)	0.1082 (6)	0.8143 (9)	0.031 (3)	0.124 (3)
H9A	0.7677	0.1312	0.8379	0.037*	0.124 (3)
H9B	0.8011	0.0723	0.8572	0.037*	0.124 (3)
C9A	0.84123 (7)	0.09213 (7)	0.76079 (14)	0.0307 (4)	0.876 (3)
H9AA	0.8439	0.0567	0.8053	0.037*	0.876 (3)
H9AB	0.8699	0.0906	0.7123	0.037*	0.876 (3)
C10	0.8625 (6)	0.1575 (7)	0.9401 (10)	0.041 (4)	0.124 (3)
H10A	0.8309	0.1742	0.9686	0.062*	0.124 (3)
H10B	0.8917	0.1859	0.9464	0.062*	0.124 (3)
H10C	0.8737	0.1227	0.9806	0.062*	0.124 (3)
C10A	0.81086 (8)	0.14483 (8)	0.91015 (14)	0.0353 (4)	0.876 (3)
H10D	0.7744	0.1479	0.8743	0.053*	0.876 (3)
H10E	0.8185	0.1785	0.9565	0.053*	0.876 (3)
H10F	0.8140	0.1094	0.9532	0.053*	0.876 (3)
C11	0.8928 (5)	0.1012 (5)	0.7970 (11)	0.034 (3)	0.124 (3)
H11A	0.8907	0.0644	0.8353	0.051*	0.124 (3)
H11B	0.9283	0.1185	0.8150	0.051*	0.124 (3)
H11C	0.8872	0.0941	0.7199	0.051*	0.124 (3)
C11A	0.90372 (7)	0.14202 (8)	0.88581 (15)	0.0373 (5)	0.876 (3)
H11D	0.9093	0.1769	0.9297	0.056*	0.876 (3)
H11E	0.9301	0.1409	0.8334	0.056*	0.876 (3)
H11F	0.9080	0.1077	0.9316	0.056*	0.876 (3)
C12	0.66027 (6)	0.21940 (6)	0.78018 (11)	0.0262 (3)	
H12	0.6956	0.2176	0.8245	0.031*	
C13	0.62524 (7)	0.26235 (8)	0.83185 (13)	0.0391 (4)	
H13A	0.6416	0.3008	0.8320	0.059*	
H13B	0.6217	0.2504	0.9055	0.059*	
H13C	0.5897	0.2636	0.7915	0.059*	
C14	0.63540 (9)	0.15932 (8)	0.77873 (14)	0.0457 (4)	
H14A	0.6004	0.1601	0.7365	0.069*	
H14B	0.6309	0.1473	0.8519	0.069*	
H14C	0.6589	0.1318	0.7468	0.069*	
C15	0.58259 (6)	0.28518 (7)	0.37426 (13)	0.0321 (3)	
H15A	0.5821	0.2866	0.2956	0.038*	0.840 (3)
H15B	0.5730	0.3241	0.3990	0.038*	0.840 (3)
H15C	0.5678	0.2535	0.3272	0.038*	0.160 (3)
H15D	0.5831	0.3210	0.3314	0.038*	0.160 (3)
C16	0.5485 (4)	0.2937 (4)	0.4666 (9)	0.034 (3)	0.160 (3)
H16A	0.5130	0.3097	0.4402	0.041*	0.160 (3)
H16B	0.5663	0.3209	0.5196	0.041*	0.160 (3)
C16A	0.54003 (6)	0.24076 (8)	0.40459 (14)	0.0281 (4)	0.840 (3)
H16C	0.5039	0.2543	0.3767	0.034*	0.840 (3)
H16D	0.5464	0.2030	0.3708	0.034*	0.840 (3)
C17	0.5200 (4)	0.1977 (5)	0.4088 (10)	0.045 (3)	0.160 (3)
H17A	0.4876	0.2165	0.3751	0.067*	0.160 (3)
H17B	0.5117	0.1577	0.4274	0.067*	0.160 (3)
H17C	0.5477	0.1976	0.3591	0.067*	0.160 (3)
C17A	0.50373 (8)	0.18920 (12)	0.5459 (2)	0.0472 (6)	0.840 (3)
H17D	0.4672	0.2023	0.5230	0.071*	0.840 (3)
H17E	0.5077	0.1831	0.6233	0.071*	0.840 (3)
H17F	0.5107	0.1528	0.5097	0.071*	0.840 (3)
C18	0.5067 (4)	0.2340 (7)	0.5861 (11)	0.053 (4)	0.160 (3)
H18A	0.4710	0.2392	0.5480	0.079*	0.160 (3)
H18B	0.5144	0.2659	0.6365	0.079*	0.160 (3)
H18C	0.5079	0.1972	0.6252	0.079*	0.160 (3)
C18A	0.52902 (8)	0.28778 (10)	0.57243 (17)	0.0422 (6)	0.840 (3)
H18D	0.5575	0.3162	0.5661	0.063*	0.840 (3)
H18E	0.5259	0.2799	0.6481	0.063*	0.840 (3)
H18F	0.4950	0.3031	0.5389	0.063*	0.840 (3)
C19	0.66622 (6)	0.39575 (6)	0.63752 (12)	0.0283 (3)	
H19	0.6379	0.3669	0.6507	0.034*	
C20	0.64160 (8)	0.43798 (8)	0.55461 (14)	0.0442 (4)	
H20A	0.6687	0.4661	0.5377	0.066*	
H20B	0.6117	0.4583	0.5827	0.066*	
H20C	0.6284	0.4169	0.4897	0.066*	
C21	0.68280 (7)	0.42570 (7)	0.74270 (13)	0.0363 (4)	
H21A	0.6979	0.3972	0.7950	0.054*	
H21B	0.6514	0.4440	0.7689	0.054*	
H21C	0.7098	0.4553	0.7322	0.054*	
Li1	0.75967 (9)	0.39092 (9)	0.50866 (19)	0.0234 (5)	
Li6	0.80947 (9)	0.28752 (9)	0.59280 (17)	0.0224 (4)	
Li5	0.82290 (9)	0.20557 (10)	0.71279 (18)	0.0234 (5)	
Li4	0.71915 (9)	0.19472 (10)	0.60043 (17)	0.0221 (4)	
Li3	0.62719 (9)	0.22294 (10)	0.54705 (19)	0.0240 (5)	
Li2	0.69456 (9)	0.31260 (10)	0.48299 (17)	0.0227 (4)	
Li7	0.72391 (9)	0.29414 (10)	0.6734 (2)	0.0261 (5)	

### Hexakis[µ3-2-(dimethylamino)ethanolato]hexa-µ2-isopropanolato-µ4-oxido-tetradecalithium(I) (1) Atomic displacement parameters (Å^2^)

**Table:**

	*U*^11^	*U*^22^	*U*^33^	*U*^12^	*U*^13^	*U*^23^
O1	0.0182 (4)	0.0153 (4)	0.0209 (4)	−0.0017 (3)	−0.0002 (3)	−0.0003 (3)
O6	0.0251 (5)	0.0212 (5)	0.0136 (4)	0.0001 (3)	−0.0013 (3)	−0.0035 (3)
O5	0.0219 (4)	0.0150 (4)	0.0183 (4)	0.0013 (3)	−0.0016 (3)	0.0035 (3)
O4	0.0219 (4)	0.0250 (5)	0.0169 (4)	−0.0008 (4)	0.0056 (3)	−0.0012 (4)
O3	0.0153 (4)	0.0202 (4)	0.0214 (5)	0.0025 (3)	−0.0017 (3)	−0.0003 (3)
O2	0.0235 (5)	0.0173 (4)	0.0216 (5)	0.0041 (3)	0.0030 (4)	−0.0043 (3)
O7	0.0112 (5)	0.0093 (5)	0.0117 (5)	−0.0002 (4)	−0.0013 (4)	0.0015 (4)
N1	0.0291 (6)	0.0165 (5)	0.0375 (7)	0.0011 (4)	0.0003 (5)	−0.0025 (5)
N3	0.0280 (6)	0.0243 (6)	0.0207 (6)	−0.0012 (5)	−0.0059 (5)	0.0036 (4)
N2	0.0179 (5)	0.0384 (7)	0.0406 (7)	0.0006 (5)	0.0031 (5)	0.0023 (6)
C1	0.038 (4)	0.031 (3)	0.043 (4)	−0.006 (3)	−0.003 (3)	0.010 (3)
C1A	0.0487 (13)	0.0195 (10)	0.0708 (19)	0.0016 (9)	−0.0044 (12)	0.0058 (10)
C2	0.049 (4)	0.020 (3)	0.088 (7)	−0.004 (3)	0.019 (4)	−0.018 (4)
C2A	0.0552 (14)	0.0368 (12)	0.0400 (13)	−0.0106 (10)	0.0078 (10)	−0.0160 (10)
C3	0.036 (3)	0.021 (3)	0.038 (4)	−0.006 (2)	−0.010 (3)	0.003 (2)
C3A	0.0308 (10)	0.0198 (9)	0.0387 (11)	−0.0062 (7)	0.0068 (8)	0.0008 (7)
C4	0.0185 (6)	0.0209 (7)	0.0474 (9)	−0.0035 (5)	−0.0007 (6)	−0.0027 (6)
C5	0.0288 (11)	0.0494 (17)	0.0341 (18)	−0.0035 (11)	−0.0074 (11)	−0.0121 (11)
C5A	0.081 (14)	0.12 (2)	0.031 (8)	−0.055 (15)	−0.010 (8)	−0.001 (10)
C6	0.0205 (15)	0.0230 (10)	0.0186 (10)	0.0006 (8)	−0.0046 (9)	−0.0039 (7)
C6A	0.030 (8)	0.114 (17)	0.020 (7)	0.012 (9)	−0.010 (5)	−0.032 (9)
C7	0.0511 (9)	0.0352 (8)	0.0197 (7)	−0.0015 (7)	0.0036 (6)	−0.0070 (6)
C8	0.0429 (8)	0.0177 (6)	0.0274 (7)	−0.0046 (6)	−0.0111 (6)	0.0048 (5)
C9	0.033 (6)	0.039 (7)	0.020 (6)	−0.006 (5)	−0.007 (4)	0.009 (5)
C9A	0.0400 (10)	0.0199 (8)	0.0291 (9)	0.0055 (6)	−0.0114 (7)	0.0026 (6)
C10	0.040 (7)	0.052 (8)	0.029 (7)	0.017 (6)	−0.009 (5)	0.005 (6)
C10A	0.0429 (10)	0.0367 (10)	0.0265 (9)	−0.0038 (8)	0.0041 (7)	0.0039 (7)
C11	0.028 (6)	0.026 (6)	0.048 (8)	0.009 (5)	0.001 (5)	0.005 (5)
C11A	0.0345 (9)	0.0392 (10)	0.0345 (10)	−0.0037 (7)	−0.0145 (7)	0.0107 (8)
C12	0.0276 (7)	0.0323 (7)	0.0193 (6)	−0.0045 (6)	0.0063 (5)	−0.0001 (5)
C13	0.0394 (9)	0.0533 (10)	0.0265 (8)	0.0036 (7)	0.0132 (6)	−0.0043 (7)
C14	0.0675 (12)	0.0387 (9)	0.0326 (9)	−0.0195 (8)	0.0128 (8)	0.0034 (7)
C15	0.0212 (7)	0.0430 (9)	0.0314 (8)	0.0092 (6)	−0.0004 (6)	0.0118 (6)
C16	0.017 (4)	0.035 (5)	0.050 (6)	0.008 (3)	0.000 (4)	−0.004 (4)
C16A	0.0165 (7)	0.0351 (10)	0.0316 (9)	0.0001 (7)	−0.0023 (6)	−0.0002 (7)
C17	0.030 (5)	0.044 (6)	0.057 (7)	−0.009 (5)	−0.012 (5)	−0.001 (5)
C17A	0.0232 (9)	0.0605 (16)	0.0589 (14)	−0.0044 (9)	0.0087 (9)	0.0175 (12)
C18	0.027 (5)	0.069 (9)	0.064 (8)	−0.012 (5)	0.008 (5)	0.006 (7)
C18A	0.0273 (9)	0.0589 (14)	0.0398 (11)	0.0102 (9)	0.0002 (8)	−0.0107 (10)
C19	0.0294 (7)	0.0263 (7)	0.0294 (7)	0.0068 (6)	0.0039 (6)	−0.0073 (6)
C20	0.0520 (10)	0.0417 (9)	0.0372 (9)	0.0259 (8)	−0.0040 (8)	−0.0066 (7)
C21	0.0456 (9)	0.0333 (8)	0.0304 (8)	0.0093 (7)	0.0062 (7)	−0.0091 (6)
Li1	0.0245 (11)	0.0174 (10)	0.0281 (12)	0.0001 (8)	0.0019 (9)	−0.0027 (9)
Li6	0.0289 (11)	0.0205 (10)	0.0180 (10)	0.0015 (9)	0.0032 (9)	0.0004 (8)
Li5	0.0270 (11)	0.0220 (11)	0.0201 (11)	−0.0004 (9)	−0.0027 (9)	0.0011 (8)
Li4	0.0220 (10)	0.0251 (11)	0.0188 (10)	0.0048 (9)	0.0006 (8)	−0.0018 (9)
Li3	0.0204 (10)	0.0255 (11)	0.0259 (11)	0.0005 (8)	0.0015 (9)	0.0004 (9)
Li2	0.0251 (11)	0.0224 (11)	0.0206 (11)	−0.0021 (8)	0.0028 (8)	−0.0036 (8)
Li7	0.0220 (11)	0.0235 (11)	0.0320 (12)	0.0001 (9)	−0.0007 (9)	0.0040 (9)

### Hexakis[µ3-2-(dimethylamino)ethanolato]hexa-µ2-isopropanolato-µ4-oxido-tetradecalithium(I) (1) Geometric parameters (Å, º)

**Table:**

O1—C4	1.3909 (16)	C8—H8AB	0.9900
O1—Li1	1.989 (2)	C8—H8BC	0.9900
O1—Li6	1.893 (2)	C8—H8BD	0.9900
O1—Li4^i^	1.974 (2)	C8—C9	1.550 (12)
O1—Li3^i^	2.136 (2)	C8—C9A	1.539 (2)
O6—C6	1.399 (3)	C9—H9A	0.9900
O6—C6A	1.476 (15)	C9—H9B	0.9900
O6—Li6	1.910 (2)	C9A—H9AA	0.9900
O6—Li5	1.888 (2)	C9A—H9AB	0.9900
O6—Li7	1.895 (2)	C10—H10A	0.9800
O5—C8	1.3934 (16)	C10—H10B	0.9800
O5—Li1^i^	2.124 (2)	C10—H10C	0.9800
O5—Li5	1.997 (2)	C10A—H10D	0.9800
O5—Li4	1.899 (2)	C10A—H10E	0.9800
O5—Li2^i^	1.973 (2)	C10A—H10F	0.9800
O4—C12	1.4048 (16)	C11—H11A	0.9800
O4—Li4	1.918 (2)	C11—H11B	0.9800
O4—Li3	1.886 (2)	C11—H11C	0.9800
O4—Li7	1.900 (2)	C11A—H11D	0.9800
O3—C15	1.3911 (16)	C11A—H11E	0.9800
O3—Li6^i^	1.981 (2)	C11A—H11F	0.9800
O3—Li5^i^	2.132 (3)	C12—H12	1.0000
O3—Li3	1.989 (2)	C12—C13	1.515 (2)
O3—Li2	1.901 (2)	C12—C14	1.521 (2)
O2—C19	1.4017 (16)	C13—H13A	0.9800
O2—Li1	1.901 (2)	C13—H13B	0.9800
O2—Li2	1.920 (2)	C13—H13C	0.9800
O2—Li7	1.898 (2)	C14—H14A	0.9800
O7—Li6	1.997 (2)	C14—H14B	0.9800
O7—Li6^i^	1.998 (2)	C14—H14C	0.9800
O7—Li4^i^	2.007 (2)	C15—H15A	0.9900
O7—Li4	2.007 (2)	C15—H15B	0.9900
O7—Li2	2.003 (2)	C15—H15C	0.9900
O7—Li2^i^	2.003 (2)	C15—H15D	0.9900
O7—Li7	2.554 (3)	C15—C16	1.524 (10)
O7—Li7^i^	2.554 (3)	C15—C16A	1.556 (2)
N1—C1	1.556 (7)	C16—H16A	0.9900
N1—C1A	1.450 (2)	C16—H16B	0.9900
N1—C2	1.415 (7)	C16A—H16C	0.9900
N1—C2A	1.496 (3)	C16A—H16D	0.9900
N1—C3	1.435 (6)	C17—H17A	0.9800
N1—C3A	1.448 (2)	C17—H17B	0.9800
N1—Li1	2.157 (2)	C17—H17C	0.9800
N3—C9	1.520 (12)	C17A—H17D	0.9800
N3—C9A	1.460 (2)	C17A—H17E	0.9800
N3—C10	1.435 (13)	C17A—H17F	0.9800
N3—C10A	1.468 (2)	C18—H18A	0.9800
N3—C11	1.542 (12)	C18—H18B	0.9800
N3—C11A	1.471 (2)	C18—H18C	0.9800
N3—Li5	2.125 (2)	C18A—H18D	0.9800
N2—C16	1.554 (11)	C18A—H18E	0.9800
N2—C16A	1.436 (2)	C18A—H18F	0.9800
N2—C17	1.652 (11)	C19—H19	1.0000
N2—C17A	1.469 (2)	C19—C20	1.514 (2)
N2—C18	1.294 (12)	C19—C21	1.514 (2)
N2—C18A	1.484 (3)	C20—H20A	0.9800
N2—Li3	2.141 (3)	C20—H20B	0.9800
C1—H1A	0.9800	C20—H20C	0.9800
C1—H1B	0.9800	C21—H21A	0.9800
C1—H1C	0.9800	C21—H21B	0.9800
C1A—H1AA	0.9800	C21—H21C	0.9800
C1A—H1AB	0.9800	Li1—O5^i^	2.124 (2)
C1A—H1AC	0.9800	Li1—C8^i^	2.716 (3)
C2—H2A	0.9800	Li1—Li6	2.850 (3)
C2—H2B	0.9800	Li1—Li4^i^	2.496 (3)
C2—H2C	0.9800	Li1—Li2	2.437 (3)
C2A—H2AA	0.9800	Li1—Li7	3.238 (3)
C2A—H2AB	0.9800	Li6—O3^i^	1.981 (2)
C2A—H2AC	0.9800	Li6—Li5	2.425 (3)
C3—H3A	0.9900	Li6—Li4	3.128 (3)
C3—H3B	0.9900	Li6—Li4^i^	2.499 (3)
C3—C4	1.559 (7)	Li6—Li3^i^	2.500 (3)
C3A—H3AA	0.9900	Li6—Li2^i^	2.501 (3)
C3A—H3AB	0.9900	Li6—Li2	3.123 (3)
C3A—C4	1.529 (2)	Li6—Li7	2.470 (3)
C4—H4AA	0.9900	Li5—O3^i^	2.132 (3)
C4—H4AB	0.9900	Li5—C15^i^	2.719 (3)
C4—H4BC	0.9900	Li5—Li4	2.845 (3)
C4—H4BD	0.9900	Li5—Li2^i^	2.495 (3)
C5—H5A	0.9800	Li5—Li7	3.218 (3)
C5—H5B	0.9800	Li4—O1^i^	1.974 (2)
C5—H5C	0.9800	Li4—Li1^i^	2.496 (3)
C5—C6	1.533 (5)	Li4—Li6^i^	2.499 (3)
C5A—H5AA	0.9800	Li4—Li3	2.426 (3)
C5A—H5AB	0.9800	Li4—Li2	3.131 (3)
C5A—H5AC	0.9800	Li4—Li2^i^	2.505 (3)
C5A—C6A	1.32 (3)	Li4—Li7	2.473 (3)
C6—H6	1.0000	Li3—O1^i^	2.136 (2)
C6—C7	1.511 (3)	Li3—C4^i^	2.732 (3)
C6A—H6A	1.0000	Li3—Li6^i^	2.500 (3)
C6A—C7	1.586 (17)	Li3—Li2	2.842 (3)
C7—H7AA	0.9800	Li3—Li7	3.216 (3)
C7—H7AB	0.9800	Li2—O5^i^	1.974 (2)
C7—H7AC	0.9800	Li2—Li6^i^	2.501 (3)
C7—H7BD	0.9800	Li2—Li5^i^	2.495 (3)
C7—H7BE	0.9800	Li2—Li4^i^	2.505 (3)
C7—H7BF	0.9800	Li2—Li7	2.472 (3)
C8—H8AA	0.9900		
			
C4—O1—Li1	107.47 (10)	O5^i^—Li1—Li4^i^	47.70 (7)
C4—O1—Li6	132.56 (11)	O5^i^—Li1—Li2	50.71 (7)
C4—O1—Li4^i^	144.17 (11)	O5^i^—Li1—Li7	98.86 (9)
C4—O1—Li3^i^	99.37 (10)	O2—Li1—O1	120.02 (12)
Li1—O1—Li3^i^	149.94 (10)	O2—Li1—O5^i^	97.57 (10)
Li6—O1—Li1	94.45 (10)	O2—Li1—N1	127.33 (12)
Li6—O1—Li4^i^	80.49 (10)	O2—Li1—C8^i^	111.50 (10)
Li6—O1—Li3^i^	76.43 (10)	O2—Li1—Li6	78.84 (9)
Li4^i^—O1—Li1	78.07 (9)	O2—Li1—Li4^i^	106.22 (11)
Li4^i^—O1—Li3^i^	72.22 (9)	O2—Li1—Li2	50.71 (8)
C6—O6—Li6	120.60 (14)	O2—Li1—Li7	31.51 (6)
C6—O6—Li5	130.56 (17)	N1—Li1—C8^i^	96.69 (9)
C6—O6—Li7	111.35 (18)	N1—Li1—Li6	119.00 (11)
C6A—O6—Li6	124.5 (9)	N1—Li1—Li4^i^	125.00 (12)
C6A—O6—Li5	107.9 (13)	N1—Li1—Li2	169.02 (13)
C6A—O6—Li7	132.5 (11)	N1—Li1—Li7	136.48 (11)
Li5—O6—Li6	79.35 (10)	C8^i^—Li1—Li6	126.15 (10)
Li5—O6—Li7	116.55 (11)	C8^i^—Li1—Li7	125.11 (9)
Li7—O6—Li6	80.97 (11)	Li6—Li1—Li7	47.35 (7)
C8—O5—Li1^i^	99.01 (10)	Li4^i^—Li1—C8^i^	71.54 (8)
C8—O5—Li5	107.22 (10)	Li4^i^—Li1—Li6	55.26 (8)
C8—O5—Li4	135.38 (11)	Li4^i^—Li1—Li7	83.86 (9)
C8—O5—Li2^i^	141.39 (11)	Li2—Li1—C8^i^	76.08 (9)
Li5—O5—Li1^i^	150.26 (10)	Li2—Li1—Li6	71.92 (9)
Li4—O5—Li1^i^	76.48 (9)	Li2—Li1—Li4^i^	61.03 (9)
Li4—O5—Li5	93.77 (10)	Li2—Li1—Li7	49.21 (8)
Li4—O5—Li2^i^	80.60 (10)	O1—Li6—O6	136.07 (13)
Li2^i^—O5—Li1^i^	72.91 (9)	O1—Li6—O3^i^	104.34 (11)
Li2^i^—O5—Li5	77.84 (10)	O1—Li6—O7	101.74 (10)
C12—O4—Li4	119.48 (11)	O1—Li6—Li1	44.09 (7)
C12—O4—Li3	128.30 (11)	O1—Li6—Li5	157.33 (13)
C12—O4—Li7	114.21 (11)	O1—Li6—Li4^i^	51.18 (8)
Li3—O4—Li4	79.25 (10)	O1—Li6—Li4	139.52 (11)
Li3—O4—Li7	116.33 (11)	O1—Li6—Li3^i^	56.17 (8)
Li7—O4—Li4	80.73 (11)	O1—Li6—Li2	83.14 (9)
C15—O3—Li6^i^	140.30 (11)	O1—Li6—Li2^i^	118.32 (12)
C15—O3—Li5^i^	98.87 (10)	O1—Li6—Li7	118.50 (12)
C15—O3—Li3	107.53 (10)	O6—Li6—O3^i^	102.40 (11)
C15—O3—Li2	136.53 (12)	O6—Li6—O7	107.75 (11)
Li6^i^—O3—Li5^i^	72.14 (9)	O6—Li6—Li1	108.38 (10)
Li6^i^—O3—Li3	78.04 (10)	O6—Li6—Li5	49.93 (8)
Li3—O3—Li5^i^	149.72 (10)	O6—Li6—Li4	71.73 (8)
Li2—O3—Li6^i^	80.18 (10)	O6—Li6—Li4^i^	151.59 (13)
Li2—O3—Li5^i^	76.19 (9)	O6—Li6—Li3^i^	149.98 (13)
Li2—O3—Li3	93.82 (10)	O6—Li6—Li2^i^	105.53 (11)
C19—O2—Li1	128.68 (11)	O6—Li6—Li2	100.11 (10)
C19—O2—Li2	119.00 (11)	O6—Li6—Li7	49.26 (8)
C19—O2—Li7	113.39 (11)	O3^i^—Li6—O7	98.98 (10)
Li1—O2—Li2	79.26 (10)	O3^i^—Li6—Li1	147.08 (12)
Li7—O2—Li1	116.92 (11)	O3^i^—Li6—Li5	56.81 (8)
Li7—O2—Li2	80.72 (11)	O3^i^—Li6—Li4^i^	100.34 (10)
Li6—O7—Li6^i^	180.0	O3^i^—Li6—Li4	93.21 (9)
Li6^i^—O7—Li4^i^	102.76 (9)	O3^i^—Li6—Li3^i^	51.13 (8)
Li6—O7—Li4^i^	77.24 (9)	O3^i^—Li6—Li2	136.91 (11)
Li6^i^—O7—Li4	77.25 (9)	O3^i^—Li6—Li2^i^	48.51 (7)
Li6—O7—Li4	102.75 (9)	O3^i^—Li6—Li7	136.86 (12)
Li6—O7—Li2^i^	77.38 (9)	O7—Li6—Li1	82.71 (9)
Li6^i^—O7—Li2^i^	102.62 (9)	O7—Li6—Li5	94.23 (10)
Li6^i^—O7—Li2	77.38 (9)	O7—Li6—Li4^i^	51.54 (7)
Li6—O7—Li2	102.62 (9)	O7—Li6—Li4	38.73 (6)
Li6—O7—Li7	64.39 (8)	O7—Li6—Li3^i^	92.10 (10)
Li6^i^—O7—Li7^i^	64.39 (8)	O7—Li6—Li2^i^	51.41 (7)
Li6—O7—Li7^i^	115.61 (8)	O7—Li6—Li2	38.76 (6)
Li6^i^—O7—Li7	115.62 (8)	O7—Li6—Li7	68.80 (9)
Li4—O7—Li4^i^	180.0	Li1—Li6—Li4	107.12 (9)
Li4—O7—Li7	64.36 (8)	Li1—Li6—Li2	47.90 (7)
Li4^i^—O7—Li7^i^	64.36 (8)	Li5—Li6—Li1	156.11 (12)
Li4—O7—Li7^i^	115.64 (8)	Li5—Li6—Li4^i^	137.96 (12)
Li4^i^—O7—Li7	115.64 (8)	Li5—Li6—Li4	60.06 (8)
Li2^i^—O7—Li4	77.34 (9)	Li5—Li6—Li3^i^	107.76 (11)
Li2—O7—Li4^i^	77.34 (9)	Li5—Li6—Li2	118.99 (11)
Li2—O7—Li4	102.66 (9)	Li5—Li6—Li2^i^	60.84 (9)
Li2^i^—O7—Li4^i^	102.66 (9)	Li5—Li6—Li7	82.19 (10)
Li2—O7—Li2^i^	180.0	Li4^i^—Li6—Li1	55.16 (8)
Li2—O7—Li7	64.39 (8)	Li4^i^—Li6—Li4	90.27 (9)
Li2^i^—O7—Li7	115.61 (8)	Li4^i^—Li6—Li3^i^	58.07 (9)
Li2—O7—Li7^i^	115.61 (8)	Li4^i^—Li6—Li2^i^	77.53 (10)
Li2^i^—O7—Li7^i^	64.39 (8)	Li4^i^—Li6—Li2	51.48 (8)
Li7—O7—Li7^i^	180.0	Li3^i^—Li6—Li1	96.05 (10)
C1—N1—Li1	113.7 (3)	Li3^i^—Li6—Li4	117.99 (10)
C1A—N1—C2A	108.45 (17)	Li3^i^—Li6—Li2^i^	69.26 (9)
C1A—N1—Li1	124.52 (14)	Li3^i^—Li6—Li2	109.29 (10)
C2—N1—C1	105.7 (5)	Li2^i^—Li6—Li1	129.38 (11)
C2—N1—C3	117.0 (5)	Li2—Li6—Li4	60.11 (7)
C2—N1—Li1	118.2 (3)	Li2^i^—Li6—Li4	51.39 (8)
C2A—N1—Li1	105.61 (13)	Li2^i^—Li6—Li2	90.17 (9)
C3—N1—C1	108.6 (4)	Li7—Li6—Li1	74.59 (9)
C3—N1—Li1	93.4 (3)	Li7—Li6—Li4	50.77 (8)
C3A—N1—C1A	110.50 (16)	Li7—Li6—Li4^i^	102.33 (11)
C3A—N1—C2A	109.76 (15)	Li7—Li6—Li3^i^	159.40 (13)
C3A—N1—Li1	97.07 (11)	Li7—Li6—Li2^i^	102.16 (11)
C9—N3—C11	104.8 (7)	Li7—Li6—Li2	50.85 (8)
C9—N3—Li5	93.8 (4)	O6—Li5—O5	120.97 (12)
C9A—N3—C10A	111.94 (13)	O6—Li5—O3^i^	97.77 (10)
C9A—N3—C11A	110.02 (13)	O6—Li5—N3	126.79 (12)
C9A—N3—Li5	96.89 (10)	O6—Li5—C15^i^	111.01 (11)
C10—N3—C9	111.0 (8)	O6—Li5—Li6	50.72 (8)
C10—N3—C11	108.0 (7)	O6—Li5—Li4	79.43 (9)
C10—N3—Li5	122.2 (6)	O6—Li5—Li2^i^	106.47 (11)
C10A—N3—C11A	108.23 (13)	O6—Li5—Li7	31.79 (6)
C10A—N3—Li5	105.93 (12)	O5—Li5—O3^i^	95.91 (10)
C11—N3—Li5	114.8 (5)	O5—Li5—N3	88.55 (10)
C11A—N3—Li5	123.37 (12)	O5—Li5—C15^i^	109.28 (10)
C16—N2—C17	98.1 (6)	O5—Li5—Li6	102.37 (11)
C16—N2—Li3	91.7 (3)	O5—Li5—Li4	41.77 (7)
C16A—N2—C17A	111.09 (15)	O5—Li5—Li2^i^	50.66 (8)
C16A—N2—C18A	111.66 (15)	O5—Li5—Li7	89.32 (9)
C16A—N2—Li3	96.79 (11)	O3^i^—Li5—C15^i^	30.36 (5)
C17—N2—Li3	109.3 (4)	O3^i^—Li5—Li6	51.05 (8)
C17A—N2—C18A	107.33 (16)	O3^i^—Li5—Li4	98.38 (9)
C17A—N2—Li3	123.26 (13)	O3^i^—Li5—Li2^i^	47.74 (7)
C18—N2—C16	111.8 (8)	O3^i^—Li5—Li7	99.53 (9)
C18—N2—C17	111.1 (7)	N3—Li5—O3^i^	124.10 (12)
C18—N2—Li3	128.9 (6)	N3—Li5—C15^i^	96.26 (9)
C18A—N2—Li3	106.31 (12)	N3—Li5—Li6	168.21 (13)
N1—C1—H1A	109.5	N3—Li5—Li4	119.36 (11)
N1—C1—H1B	109.5	N3—Li5—Li2^i^	125.53 (12)
N1—C1—H1C	109.5	N3—Li5—Li7	136.30 (11)
H1A—C1—H1B	109.5	C15^i^—Li5—Li4	126.71 (10)
H1A—C1—H1C	109.5	C15^i^—Li5—Li7	125.37 (9)
H1B—C1—H1C	109.5	Li6—Li5—C15^i^	76.08 (9)
N1—C1A—H1AA	109.5	Li6—Li5—Li4	72.34 (9)
N1—C1A—H1AB	109.5	Li6—Li5—Li2^i^	61.09 (9)
N1—C1A—H1AC	109.5	Li6—Li5—Li7	49.52 (8)
H1AA—C1A—H1AB	109.5	Li4—Li5—Li7	47.65 (7)
H1AA—C1A—H1AC	109.5	Li2^i^—Li5—C15^i^	71.84 (9)
H1AB—C1A—H1AC	109.5	Li2^i^—Li5—Li4	55.50 (8)
N1—C2—H2A	109.5	Li2^i^—Li5—Li7	84.22 (9)
N1—C2—H2B	109.5	O1^i^—Li4—O7	98.61 (10)
N1—C2—H2C	109.5	O1^i^—Li4—Li1^i^	51.22 (8)
H2A—C2—H2B	109.5	O1^i^—Li4—Li6^i^	48.32 (7)
H2A—C2—H2C	109.5	O1^i^—Li4—Li6	136.33 (11)
H2B—C2—H2C	109.5	O1^i^—Li4—Li5	147.41 (11)
N1—C2A—H2AA	109.5	O1^i^—Li4—Li3	56.97 (8)
N1—C2A—H2AB	109.5	O1^i^—Li4—Li2^i^	100.16 (10)
N1—C2A—H2AC	109.5	O1^i^—Li4—Li2	92.91 (9)
H2AA—C2A—H2AB	109.5	O1^i^—Li4—Li7	136.74 (12)
H2AA—C2A—H2AC	109.5	O5—Li4—O1^i^	104.42 (11)
H2AB—C2A—H2AC	109.5	O5—Li4—O4	136.55 (13)
N1—C3—H3A	109.3	O5—Li4—O7	101.31 (10)
N1—C3—H3B	109.3	O5—Li4—Li1^i^	55.81 (8)
N1—C3—C4	111.7 (4)	O5—Li4—Li6^i^	117.89 (11)
H3A—C3—H3B	107.9	O5—Li4—Li6	82.99 (9)
C4—C3—H3A	109.3	O5—Li4—Li5	44.47 (7)
C4—C3—H3B	109.3	O5—Li4—Li3	157.77 (14)
N1—C3A—H3AA	109.1	O5—Li4—Li2	139.03 (11)
N1—C3A—H3AB	109.1	O5—Li4—Li2^i^	51.00 (8)
N1—C3A—C4	112.69 (14)	O5—Li4—Li7	118.42 (12)
H3AA—C3A—H3AB	107.8	O4—Li4—O1^i^	102.54 (11)
C4—C3A—H3AA	109.1	O4—Li4—O7	107.59 (11)
C4—C3A—H3AB	109.1	O4—Li4—Li1^i^	150.02 (13)
O1—C4—C3	110.1 (3)	O4—Li4—Li6	100.02 (10)
O1—C4—C3A	112.50 (12)	O4—Li4—Li6^i^	105.51 (11)
O1—C4—H4AA	109.1	O4—Li4—Li5	108.24 (10)
O1—C4—H4AB	109.1	O4—Li4—Li3	49.79 (8)
O1—C4—H4BC	109.6	O4—Li4—Li2	71.67 (8)
O1—C4—H4BD	109.6	O4—Li4—Li2^i^	151.27 (13)
C3—C4—H4BC	109.6	O4—Li4—Li7	49.31 (8)
C3—C4—H4BD	109.6	O7—Li4—Li1^i^	92.35 (10)
C3A—C4—H4AA	109.1	O7—Li4—Li6^i^	51.22 (7)
C3A—C4—H4AB	109.1	O7—Li4—Li6	38.52 (6)
H4AA—C4—H4AB	107.8	O7—Li4—Li5	82.26 (9)
H4BC—C4—H4BD	108.2	O7—Li4—Li3	94.07 (10)
H5A—C5—H5B	109.5	O7—Li4—Li2^i^	51.27 (7)
H5A—C5—H5C	109.5	O7—Li4—Li2	38.63 (6)
H5B—C5—H5C	109.5	O7—Li4—Li7	68.63 (9)
C6—C5—H5A	109.5	Li1^i^—Li4—Li6^i^	69.57 (9)
C6—C5—H5B	109.5	Li1^i^—Li4—Li6	109.36 (10)
C6—C5—H5C	109.5	Li1^i^—Li4—Li5	96.22 (10)
H5AA—C5A—H5AB	109.5	Li1^i^—Li4—Li2	118.21 (10)
H5AA—C5A—H5AC	109.5	Li1^i^—Li4—Li2^i^	58.33 (9)
H5AB—C5A—H5AC	109.5	Li6^i^—Li4—Li6	89.73 (9)
C6A—C5A—H5AA	109.5	Li6^i^—Li4—Li5	128.89 (11)
C6A—C5A—H5AB	109.5	Li6—Li4—Li2	59.86 (7)
C6A—C5A—H5AC	109.5	Li6^i^—Li4—Li2	51.26 (8)
O6—C6—C5	110.6 (2)	Li6^i^—Li4—Li2^i^	77.21 (10)
O6—C6—H6	108.5	Li5—Li4—Li6	47.60 (7)
O6—C6—C7	111.54 (19)	Li5—Li4—Li2	106.55 (9)
C5—C6—H6	108.5	Li3—Li4—Li1^i^	108.06 (11)
C7—C6—C5	109.2 (3)	Li3—Li4—Li6^i^	60.97 (9)
C7—C6—H6	108.5	Li3—Li4—Li6	118.61 (11)
O6—C6A—H6A	102.8	Li3—Li4—Li5	155.59 (12)
O6—C6A—C7	103.5 (10)	Li3—Li4—Li2	59.93 (8)
C5A—C6A—O6	118 (2)	Li3—Li4—Li2^i^	137.72 (12)
C5A—C6A—H6A	102.8	Li3—Li4—Li7	82.07 (10)
C5A—C6A—C7	124 (3)	Li2^i^—Li4—Li6	51.26 (8)
C7—C6A—H6A	102.8	Li2^i^—Li4—Li5	55.14 (8)
C6—C7—H7AA	109.5	Li2^i^—Li4—Li2	89.90 (10)
C6—C7—H7AB	109.5	Li7—Li4—Li1^i^	159.39 (13)
C6—C7—H7AC	109.5	Li7—Li4—Li6^i^	101.98 (11)
C6A—C7—H7BD	109.5	Li7—Li4—Li6	50.70 (8)
C6A—C7—H7BE	109.5	Li7—Li4—Li5	74.10 (9)
C6A—C7—H7BF	109.5	Li7—Li4—Li2^i^	101.96 (11)
H7AA—C7—H7AB	109.5	Li7—Li4—Li2	50.72 (8)
H7AA—C7—H7AC	109.5	O1^i^—Li3—N2	125.00 (12)
H7AB—C7—H7AC	109.5	O1^i^—Li3—C4^i^	30.15 (5)
H7BD—C7—H7BE	109.5	O1^i^—Li3—Li6^i^	47.40 (7)
H7BD—C7—H7BF	109.5	O1^i^—Li3—Li4	50.80 (7)
H7BE—C7—H7BF	109.5	O1^i^—Li3—Li2	98.00 (10)
O5—C8—H8AA	109.1	O1^i^—Li3—Li7	99.28 (9)
O5—C8—H8AB	109.1	O4—Li3—O1^i^	97.86 (10)
O5—C8—H8BC	109.8	O4—Li3—O3	121.25 (12)
O5—C8—H8BD	109.8	O4—Li3—N2	126.29 (13)
O5—C8—C9	109.3 (5)	O4—Li3—C4^i^	112.92 (11)
O5—C8—C9A	112.35 (12)	O4—Li3—Li6^i^	106.53 (11)
H8AA—C8—H8AB	107.9	O4—Li3—Li4	50.95 (8)
H8BC—C8—H8BD	108.3	O4—Li3—Li2	79.63 (9)
C9—C8—H8BC	109.8	O4—Li3—Li7	31.97 (6)
C9—C8—H8BD	109.8	O3—Li3—O1^i^	95.70 (10)
C9A—C8—H8AA	109.1	O3—Li3—N2	88.05 (10)
C9A—C8—H8AB	109.1	O3—Li3—C4^i^	106.95 (10)
N3—C9—C8	108.1 (7)	O3—Li3—Li6^i^	50.83 (8)
N3—C9—H9A	110.1	O3—Li3—Li4	102.60 (11)
N3—C9—H9B	110.1	O3—Li3—Li2	41.88 (7)
C8—C9—H9A	110.1	O3—Li3—Li7	89.45 (9)
C8—C9—H9B	110.1	N2—Li3—C4^i^	96.68 (9)
H9A—C9—H9B	108.4	N2—Li3—Li6^i^	125.79 (12)
N3—C9A—C8	111.94 (13)	N2—Li3—Li4	168.66 (13)
N3—C9A—H9AA	109.2	N2—Li3—Li2	118.76 (11)
N3—C9A—H9AB	109.2	N2—Li3—Li7	135.68 (11)
C8—C9A—H9AA	109.2	C4^i^—Li3—Li2	125.18 (10)
C8—C9A—H9AB	109.2	C4^i^—Li3—Li7	126.16 (9)
H9AA—C9A—H9AB	107.9	Li6^i^—Li3—C4^i^	70.17 (8)
N3—C10—H10A	109.5	Li6^i^—Li3—Li2	55.39 (8)
N3—C10—H10B	109.5	Li6^i^—Li3—Li7	84.05 (9)
N3—C10—H10C	109.5	Li4—Li3—C4^i^	76.63 (9)
H10A—C10—H10B	109.5	Li4—Li3—Li6^i^	60.96 (9)
H10A—C10—H10C	109.5	Li4—Li3—Li2	72.44 (9)
H10B—C10—H10C	109.5	Li4—Li3—Li7	49.59 (8)
N3—C10A—H10D	109.5	Li2—Li3—Li7	47.68 (7)
N3—C10A—H10E	109.5	O5^i^—Li2—O7	98.88 (10)
N3—C10A—H10F	109.5	O5^i^—Li2—Li1	56.39 (8)
H10D—C10A—H10E	109.5	O5^i^—Li2—Li6	93.01 (9)
H10D—C10A—H10F	109.5	O5^i^—Li2—Li6^i^	100.47 (10)
H10E—C10A—H10F	109.5	O5^i^—Li2—Li5^i^	51.50 (8)
N3—C11—H11A	109.5	O5^i^—Li2—Li4	136.79 (11)
N3—C11—H11B	109.5	O5^i^—Li2—Li4^i^	48.40 (7)
N3—C11—H11C	109.5	O5^i^—Li2—Li3	147.60 (12)
H11A—C11—H11B	109.5	O5^i^—Li2—Li7	136.72 (12)
H11A—C11—H11C	109.5	O3—Li2—O5^i^	104.67 (11)
H11B—C11—H11C	109.5	O3—Li2—O2	136.40 (13)
N3—C11A—H11D	109.5	O3—Li2—O7	101.53 (10)
N3—C11A—H11E	109.5	O3—Li2—Li1	157.39 (13)
N3—C11A—H11F	109.5	O3—Li2—Li6	139.21 (11)
H11D—C11A—H11E	109.5	O3—Li2—Li6^i^	51.31 (8)
H11D—C11A—H11F	109.5	O3—Li2—Li5^i^	56.07 (8)
H11E—C11A—H11F	109.5	O3—Li2—Li4^i^	118.28 (11)
O4—C12—H12	107.9	O3—Li2—Li4	82.92 (9)
O4—C12—C13	111.33 (12)	O3—Li2—Li3	44.30 (7)
O4—C12—C14	111.17 (12)	O3—Li2—Li7	118.26 (12)
C13—C12—H12	107.9	O2—Li2—O5^i^	102.24 (11)
C13—C12—C14	110.35 (14)	O2—Li2—O7	107.46 (11)
C14—C12—H12	107.9	O2—Li2—Li1	50.03 (8)
C12—C13—H13A	109.5	O2—Li2—Li6^i^	151.18 (13)
C12—C13—H13B	109.5	O2—Li2—Li6	71.54 (8)
C12—C13—H13C	109.5	O2—Li2—Li5^i^	150.30 (13)
H13A—C13—H13B	109.5	O2—Li2—Li4^i^	105.27 (11)
H13A—C13—H13C	109.5	O2—Li2—Li4	99.98 (10)
H13B—C13—H13C	109.5	O2—Li2—Li3	108.22 (11)
C12—C14—H14A	109.5	O2—Li2—Li7	49.25 (8)
C12—C14—H14B	109.5	O7—Li2—Li1	94.20 (10)
C12—C14—H14C	109.5	O7—Li2—Li6	38.62 (6)
H14A—C14—H14B	109.5	O7—Li2—Li6^i^	51.21 (7)
H14A—C14—H14C	109.5	O7—Li2—Li5^i^	91.99 (10)
H14B—C14—H14C	109.5	O7—Li2—Li4	38.71 (6)
O3—C15—H15A	109.1	O7—Li2—Li4^i^	51.39 (7)
O3—C15—H15B	109.1	O7—Li2—Li3	82.51 (9)
O3—C15—H15C	110.3	O7—Li2—Li7	68.68 (9)
O3—C15—H15D	110.3	Li1—Li2—Li6^i^	137.58 (12)
O3—C15—C16	107.0 (4)	Li1—Li2—Li6	60.18 (8)
O3—C15—C16A	112.55 (12)	Li1—Li2—Li5^i^	107.72 (11)
H15A—C15—H15B	107.8	Li1—Li2—Li4	119.08 (10)
H15C—C15—H15D	108.6	Li1—Li2—Li4^i^	60.65 (9)
C16—C15—H15C	110.3	Li1—Li2—Li3	156.00 (12)
C16—C15—H15D	110.3	Li1—Li2—Li7	82.52 (10)
C16A—C15—H15A	109.1	Li6^i^—Li2—Li6	89.83 (9)
C16A—C15—H15B	109.1	Li6^i^—Li2—Li4^i^	77.35 (10)
N2—C16—H16A	110.3	Li6^i^—Li2—Li4	51.21 (8)
N2—C16—H16B	110.3	Li6—Li2—Li4	60.03 (7)
C15—C16—N2	107.0 (6)	Li6^i^—Li2—Li3	55.35 (8)
C15—C16—H16A	110.3	Li5^i^—Li2—Li6^i^	58.07 (9)
C15—C16—H16B	110.3	Li5^i^—Li2—Li6	117.91 (10)
H16A—C16—H16B	108.6	Li5^i^—Li2—Li4^i^	69.36 (9)
N2—C16A—C15	111.46 (13)	Li5^i^—Li2—Li4	109.06 (10)
N2—C16A—H16C	109.3	Li5^i^—Li2—Li3	96.17 (10)
N2—C16A—H16D	109.3	Li4^i^—Li2—Li6	51.31 (8)
C15—C16A—H16C	109.3	Li4^i^—Li2—Li4	90.10 (9)
C15—C16A—H16D	109.3	Li4^i^—Li2—Li3	129.32 (11)
H16C—C16A—H16D	108.0	Li3—Li2—Li6	106.74 (9)
N2—C17—H17A	109.5	Li3—Li2—Li4	47.63 (7)
N2—C17—H17B	109.5	Li7—Li2—Li6^i^	101.94 (11)
N2—C17—H17C	109.5	Li7—Li2—Li6	50.79 (8)
H17A—C17—H17B	109.5	Li7—Li2—Li5^i^	159.08 (13)
H17A—C17—H17C	109.5	Li7—Li2—Li4	50.72 (8)
H17B—C17—H17C	109.5	Li7—Li2—Li4^i^	102.10 (11)
N2—C17A—H17D	109.5	Li7—Li2—Li3	74.12 (9)
N2—C17A—H17E	109.5	O6—Li7—O4	120.26 (13)
N2—C17A—H17F	109.5	O6—Li7—O2	119.52 (13)
H17D—C17A—H17E	109.5	O6—Li7—O7	89.09 (9)
H17D—C17A—H17F	109.5	O6—Li7—Li1	95.27 (10)
H17E—C17A—H17F	109.5	O6—Li7—Li6	49.77 (8)
N2—C18—H18A	109.5	O6—Li7—Li5	31.66 (6)
N2—C18—H18B	109.5	O6—Li7—Li4	89.89 (10)
N2—C18—H18C	109.5	O6—Li7—Li3	137.83 (12)
H18A—C18—H18B	109.5	O6—Li7—Li2	128.13 (13)
H18A—C18—H18C	109.5	O4—Li7—O7	89.39 (10)
H18B—C18—H18C	109.5	O4—Li7—Li1	137.71 (12)
N2—C18A—H18D	109.5	O4—Li7—Li6	128.48 (13)
N2—C18A—H18E	109.5	O4—Li7—Li5	95.74 (10)
N2—C18A—H18F	109.5	O4—Li7—Li4	49.95 (8)
H18D—C18A—H18E	109.5	O4—Li7—Li3	31.71 (7)
H18D—C18A—H18F	109.5	O4—Li7—Li2	89.88 (11)
H18E—C18A—H18F	109.5	O2—Li7—O4	120.18 (12)
O2—C19—H19	107.3	O2—Li7—O7	89.30 (10)
O2—C19—C20	111.65 (12)	O2—Li7—Li1	31.57 (6)
O2—C19—C21	111.65 (12)	O2—Li7—Li6	89.60 (11)
C20—C19—H19	107.3	O2—Li7—Li5	137.41 (12)
C20—C19—C21	111.40 (13)	O2—Li7—Li4	128.58 (13)
C21—C19—H19	107.3	O2—Li7—Li3	95.77 (10)
C19—C20—H20A	109.5	O2—Li7—Li2	50.03 (8)
C19—C20—H20B	109.5	O7—Li7—Li1	67.46 (7)
C19—C20—H20C	109.5	O7—Li7—Li5	67.48 (7)
H20A—C20—H20B	109.5	O7—Li7—Li3	67.59 (7)
H20A—C20—H20C	109.5	Li6—Li7—O7	46.81 (7)
H20B—C20—H20C	109.5	Li6—Li7—Li1	58.06 (8)
C19—C21—H21A	109.5	Li6—Li7—Li5	48.29 (8)
C19—C21—H21B	109.5	Li6—Li7—Li4	78.52 (10)
C19—C21—H21C	109.5	Li6—Li7—Li3	114.12 (11)
H21A—C21—H21B	109.5	Li6—Li7—Li2	78.36 (10)
H21A—C21—H21C	109.5	Li5—Li7—Li1	106.16 (9)
H21B—C21—H21C	109.5	Li4—Li7—O7	47.02 (7)
O1—Li1—O5^i^	96.16 (10)	Li4—Li7—Li1	114.19 (11)
O1—Li1—N1	88.06 (10)	Li4—Li7—Li5	58.25 (8)
O1—Li1—C8^i^	109.35 (10)	Li4—Li7—Li3	48.34 (8)
O1—Li1—Li6	41.46 (7)	Li3—Li7—Li1	106.29 (9)
O1—Li1—Li4^i^	50.71 (7)	Li3—Li7—Li5	106.42 (9)
O1—Li1—Li2	102.05 (10)	Li2—Li7—O7	46.93 (7)
O1—Li1—Li7	88.70 (9)	Li2—Li7—Li1	48.27 (8)
O5^i^—Li1—N1	124.63 (12)	Li2—Li7—Li5	114.11 (11)
O5^i^—Li1—C8^i^	30.44 (5)	Li2—Li7—Li4	78.55 (10)
O5^i^—Li1—Li6	97.88 (9)	Li2—Li7—Li3	58.20 (8)
			
O5—C8—C9—N3	−63.5 (8)	Li6—O6—Li5—C15^i^	49.68 (11)
O5—C8—C9A—N3	54.21 (19)	Li6—O6—Li5—Li4	−75.75 (9)
O3—C15—C16—N2	−69.0 (6)	Li6—O6—Li5—Li2^i^	−26.74 (11)
O3—C15—C16A—N2	53.89 (18)	Li6—O6—Li5—Li7	−74.19 (12)
N1—C3—C4—O1	−59.8 (5)	Li6—O6—Li7—O4	116.74 (16)
N1—C3A—C4—O1	54.81 (19)	Li6—O6—Li7—O2	−60.70 (15)
C1—N1—C3—C4	−63.4 (5)	Li6—O6—Li7—O7	27.98 (8)
C1A—N1—C3A—C4	−175.25 (16)	Li6—O6—Li7—Li1	−39.29 (9)
C2—N1—C3—C4	177.1 (5)	Li6—O6—Li7—Li5	73.23 (12)
C2A—N1—C3A—C4	65.19 (19)	Li6—O6—Li7—Li4	74.99 (10)
C4—O1—Li6—O6	53.4 (2)	Li6—O6—Li7—Li3	82.14 (18)
C4—O1—Li6—O3^i^	−72.35 (16)	Li6—O6—Li7—Li2	−0.31 (15)
C4—O1—Li6—O7	−174.92 (11)	Li6^i^—O3—C15—C16	128.4 (4)
C4—O1—Li6—Li1	118.71 (15)	Li6^i^—O3—C15—C16A	68.3 (2)
C4—O1—Li6—Li5	−41.0 (4)	Li5—O6—C6—C5	32.4 (3)
C4—O1—Li6—Li4	174.79 (14)	Li5—O6—C6—C7	−89.3 (3)
C4—O1—Li6—Li4^i^	−164.19 (15)	Li5—O6—C6A—C5A	101 (4)
C4—O1—Li6—Li3^i^	−90.33 (14)	Li5—O6—C6A—C7	−118.0 (16)
C4—O1—Li6—Li2^i^	−122.63 (15)	Li5—O6—Li7—O4	43.52 (19)
C4—O1—Li6—Li2	150.90 (12)	Li5—O6—Li7—O2	−133.93 (14)
C4—O1—Li6—Li7	112.98 (16)	Li5—O6—Li7—O7	−45.25 (12)
C6—O6—Li5—O5	158.28 (17)	Li5—O6—Li7—Li1	−112.52 (11)
C6—O6—Li5—O3^i^	−100.01 (18)	Li5—O6—Li7—Li6	−73.23 (12)
C6—O6—Li5—N3	44.0 (3)	Li5—O6—Li7—Li4	1.76 (14)
C6—O6—Li5—C15^i^	−71.7 (2)	Li5—O6—Li7—Li3	8.9 (2)
C6—O6—Li5—Li6	−121.39 (19)	Li5—O6—Li7—Li2	−73.54 (18)
C6—O6—Li5—Li4	162.86 (17)	Li5—O5—C8—C9	31.5 (5)
C6—O6—Li5—Li2^i^	−148.13 (17)	Li5—O5—C8—C9A	−26.04 (16)
C6—O6—Li5—Li7	164.4 (2)	Li5—O5—Li4—O1^i^	168.51 (11)
C6—O6—Li7—O4	−123.83 (17)	Li5—O5—Li4—O4	−64.8 (2)
C6—O6—Li7—O2	58.7 (2)	Li5—O5—Li4—O7	66.44 (11)
C6—O6—Li7—O7	147.40 (13)	Li5—O5—Li4—Li1^i^	151.54 (10)
C6—O6—Li7—Li1	80.13 (14)	Li5—O5—Li4—Li6^i^	118.35 (13)
C6—O6—Li7—Li6	119.43 (15)	Li5—O5—Li4—Li6	32.38 (9)
C6—O6—Li7—Li5	−167.35 (18)	Li5—O5—Li4—Li3	−160.6 (3)
C6—O6—Li7—Li4	−165.58 (13)	Li5—O5—Li4—Li2^i^	77.01 (10)
C6—O6—Li7—Li3	−158.43 (18)	Li5—O5—Li4—Li2	56.41 (17)
C6—O6—Li7—Li2	119.12 (18)	Li5—O5—Li4—Li7	−5.24 (14)
C6A—O6—Li5—O5	156.7 (9)	Li5^i^—O3—C15—C16	−159.0 (4)
C6A—O6—Li5—O3^i^	−101.6 (9)	Li5^i^—O3—C15—C16A	141.01 (13)
C6A—O6—Li5—N3	42.3 (9)	Li5—N3—C9—C8	53.9 (7)
C6A—O6—Li5—C15^i^	−73.3 (9)	Li5—N3—C9A—C8	−46.40 (15)
C6A—O6—Li5—Li6	−123.0 (9)	Li4^i^—O1—C4—C3	118.8 (3)
C6A—O6—Li5—Li4	161.2 (9)	Li4^i^—O1—C4—C3A	65.0 (2)
C6A—O6—Li5—Li2^i^	−149.8 (9)	Li4^i^—O1—Li6—O6	−142.38 (19)
C6A—O6—Li5—Li7	162.8 (9)	Li4^i^—O1—Li6—O3^i^	91.84 (11)
C6A—O6—Li7—O4	−114.1 (16)	Li4^i^—O1—Li6—O7	−10.73 (10)
C6A—O6—Li7—O2	68.5 (16)	Li4^i^—O1—Li6—Li1	−77.10 (10)
C6A—O6—Li7—O7	157.2 (16)	Li4^i^—O1—Li6—Li5	123.2 (3)
C6A—O6—Li7—Li1	89.9 (16)	Li4^i^—O1—Li6—Li4	−21.02 (18)
C6A—O6—Li7—Li6	129.2 (16)	Li4^i^—O1—Li6—Li3^i^	73.86 (9)
C6A—O6—Li7—Li5	−157.6 (16)	Li4^i^—O1—Li6—Li2	−44.91 (8)
C6A—O6—Li7—Li4	−155.8 (16)	Li4^i^—O1—Li6—Li2^i^	41.56 (13)
C6A—O6—Li7—Li3	−148.7 (16)	Li4^i^—O1—Li6—Li7	−82.83 (13)
C6A—O6—Li7—Li2	128.9 (16)	Li4—O5—C8—C9	−82.8 (5)
C8—O5—Li4—O1^i^	−72.21 (17)	Li4—O5—C8—C9A	−140.37 (15)
C8—O5—Li4—O4	54.5 (3)	Li4—O4—C12—C13	173.15 (12)
C8—O5—Li4—O7	−174.28 (11)	Li4—O4—C12—C14	−63.38 (17)
C8—O5—Li4—Li1^i^	−89.18 (15)	Li4—O4—Li3—O1^i^	21.02 (10)
C8—O5—Li4—Li6^i^	−122.37 (15)	Li4—O4—Li3—O3	−80.58 (14)
C8—O5—Li4—Li6	151.66 (13)	Li4—O4—Li3—N2	166.08 (16)
C8—O5—Li4—Li5	119.28 (16)	Li4—O4—Li3—C4^i^	48.23 (11)
C8—O5—Li4—Li3	−41.4 (4)	Li4—O4—Li3—Li6^i^	−26.78 (11)
C8—O5—Li4—Li2^i^	−163.71 (15)	Li4—O4—Li3—Li2	−75.73 (9)
C8—O5—Li4—Li2	175.70 (13)	Li4—O4—Li3—Li7	−73.93 (12)
C8—O5—Li4—Li7	114.04 (16)	Li4—O4—Li7—O6	−60.45 (15)
C10—N3—C9—C8	−179.4 (8)	Li4—O4—Li7—O2	116.98 (16)
C10A—N3—C9A—C8	63.89 (17)	Li4—O4—Li7—O7	28.15 (8)
C11—N3—C9—C8	−63.1 (9)	Li4—O4—Li7—Li1	82.61 (17)
C11A—N3—C9A—C8	−175.73 (14)	Li4—O4—Li7—Li6	0.11 (15)
C12—O4—Li3—O1^i^	−98.21 (14)	Li4—O4—Li7—Li5	−39.15 (9)
C12—O4—Li3—O3	160.19 (12)	Li4—O4—Li7—Li3	73.05 (12)
C12—O4—Li3—N2	46.8 (2)	Li4—O4—Li7—Li2	75.08 (10)
C12—O4—Li3—C4^i^	−71.00 (16)	Li3^i^—O1—C4—C3	−168.6 (3)
C12—O4—Li3—Li6^i^	−146.01 (12)	Li3^i^—O1—C4—C3A	137.50 (14)
C12—O4—Li3—Li4	−119.23 (14)	Li3^i^—O1—Li6—O6	143.8 (2)
C12—O4—Li3—Li2	165.04 (12)	Li3^i^—O1—Li6—O3^i^	17.99 (10)
C12—O4—Li3—Li7	166.84 (18)	Li3^i^—O1—Li6—O7	−84.59 (10)
C12—O4—Li7—O6	57.80 (18)	Li3^i^—O1—Li6—Li1	−150.96 (10)
C12—O4—Li7—O2	−124.77 (14)	Li3^i^—O1—Li6—Li5	49.3 (3)
C12—O4—Li7—O7	146.40 (9)	Li3^i^—O1—Li6—Li4	−94.88 (17)
C12—O4—Li7—Li1	−159.14 (15)	Li3^i^—O1—Li6—Li4^i^	−73.86 (9)
C12—O4—Li7—Li6	118.36 (16)	Li3^i^—O1—Li6—Li2^i^	−32.30 (12)
C12—O4—Li7—Li5	79.10 (12)	Li3^i^—O1—Li6—Li2	−118.77 (9)
C12—O4—Li7—Li4	118.25 (12)	Li3^i^—O1—Li6—Li7	−156.69 (14)
C12—O4—Li7—Li3	−168.70 (16)	Li3—O4—C12—C13	−86.86 (16)
C12—O4—Li7—Li2	−166.67 (10)	Li3—O4—C12—C14	36.61 (19)
C17—N2—C16—C15	−52.7 (7)	Li3—O4—Li7—O6	−133.50 (14)
C17A—N2—C16A—C15	−177.14 (15)	Li3—O4—Li7—O2	43.93 (19)
C18—N2—C16—C15	−169.4 (7)	Li3—O4—Li7—O7	−44.90 (12)
C18A—N2—C16A—C15	63.08 (17)	Li3—O4—Li7—Li1	9.6 (2)
C19—O2—Li7—O6	−125.50 (14)	Li3—O4—Li7—Li6	−72.93 (18)
C19—O2—Li7—O4	57.06 (18)	Li3—O4—Li7—Li5	−112.20 (11)
C19—O2—Li7—O7	145.95 (9)	Li3—O4—Li7—Li4	−73.05 (12)
C19—O2—Li7—Li1	−169.46 (15)	Li3—O4—Li7—Li2	2.03 (14)
C19—O2—Li7—Li6	−167.24 (10)	Li3—O3—C15—C16	36.1 (4)
C19—O2—Li7—Li5	−159.46 (15)	Li3—O3—C15—C16A	−23.97 (16)
C19—O2—Li7—Li4	117.83 (15)	Li3—N2—C16—C15	57.1 (5)
C19—O2—Li7—Li3	78.56 (11)	Li3—N2—C16A—C15	−47.50 (15)
C19—O2—Li7—Li2	117.68 (12)	Li2^i^—O5—C8—C9	123.5 (5)
Li1—O1—C4—C3	25.0 (3)	Li2^i^—O5—C8—C9A	65.9 (2)
Li1—O1—C4—C3A	−28.82 (17)	Li2^i^—O5—Li4—O1^i^	91.50 (11)
Li1—O1—Li6—O6	−65.27 (19)	Li2^i^—O5—Li4—O4	−141.8 (2)
Li1—O1—Li6—O3^i^	168.95 (11)	Li2^i^—O5—Li4—O7	−10.57 (9)
Li1—O1—Li6—O7	66.37 (11)	Li2^i^—O5—Li4—Li1^i^	74.54 (9)
Li1—O1—Li6—Li5	−159.7 (3)	Li2^i^—O5—Li4—Li6^i^	41.34 (13)
Li1—O1—Li6—Li4^i^	77.10 (10)	Li2^i^—O5—Li4—Li6	−44.62 (8)
Li1—O1—Li6—Li4	56.08 (17)	Li2^i^—O5—Li4—Li5	−77.01 (10)
Li1—O1—Li6—Li3^i^	150.96 (10)	Li2^i^—O5—Li4—Li3	122.4 (3)
Li1—O1—Li6—Li2	32.19 (9)	Li2^i^—O5—Li4—Li2	−20.59 (18)
Li1—O1—Li6—Li2^i^	118.66 (13)	Li2^i^—O5—Li4—Li7	−82.25 (13)
Li1—O1—Li6—Li7	−5.73 (14)	Li2—O3—C15—C16	−79.7 (4)
Li1^i^—O5—C8—C9	−162.6 (5)	Li2—O3—C15—C16A	−139.73 (15)
Li1^i^—O5—C8—C9A	139.78 (13)	Li2—O2—C19—C20	−65.70 (17)
Li1^i^—O5—Li4—O1^i^	16.96 (10)	Li2—O2—C19—C21	168.85 (12)
Li1^i^—O5—Li4—O4	143.7 (2)	Li2—O2—Li7—O6	116.82 (16)
Li1^i^—O5—Li4—O7	−85.11 (10)	Li2—O2—Li7—O4	−60.62 (15)
Li1^i^—O5—Li4—Li6	−119.16 (9)	Li2—O2—Li7—O7	28.27 (8)
Li1^i^—O5—Li4—Li6^i^	−33.19 (12)	Li2—O2—Li7—Li1	72.86 (12)
Li1^i^—O5—Li4—Li5	−151.54 (10)	Li2—O2—Li7—Li6	75.08 (10)
Li1^i^—O5—Li4—Li3	47.8 (3)	Li2—O2—Li7—Li5	82.86 (17)
Li1^i^—O5—Li4—Li2	−95.13 (17)	Li2—O2—Li7—Li4	0.15 (15)
Li1^i^—O5—Li4—Li2^i^	−74.54 (9)	Li2—O2—Li7—Li3	−39.12 (9)
Li1^i^—O5—Li4—Li7	−156.78 (14)	Li7—O6—C6—C5	−162.5 (3)
Li1—O2—C19—C20	34.14 (19)	Li7—O6—C6—C7	75.7 (2)
Li1—O2—C19—C21	−91.31 (16)	Li7—O6—C6A—C5A	−100 (3)
Li1—O2—Li7—O6	43.96 (19)	Li7—O6—C6A—C7	41 (3)
Li1—O2—Li7—O4	−133.48 (14)	Li7—O6—Li5—O5	−6.14 (19)
Li1—O2—Li7—O7	−44.59 (12)	Li7—O6—Li5—O3^i^	95.57 (12)
Li1—O2—Li7—Li6	2.22 (14)	Li7—O6—Li5—N3	−120.46 (15)
Li1—O2—Li7—Li5	10.0 (2)	Li7—O6—Li5—C15^i^	123.87 (12)
Li1—O2—Li7—Li4	−72.71 (18)	Li7—O6—Li5—Li6	74.19 (12)
Li1—O2—Li7—Li3	−111.98 (11)	Li7—O6—Li5—Li4	−1.56 (12)
Li1—O2—Li7—Li2	−72.86 (12)	Li7—O6—Li5—Li2^i^	47.45 (15)
Li1—N1—C3—C4	53.0 (4)	Li7—O4—C12—C13	80.21 (15)
Li1—N1—C3A—C4	−44.25 (16)	Li7—O4—C12—C14	−156.32 (14)
Li6—O1—C4—C3	−88.5 (3)	Li7—O4—Li3—O1^i^	94.95 (12)
Li6—O1—C4—C3A	−142.36 (15)	Li7—O4—Li3—O3	−6.65 (18)
Li6—O6—C6—C5	−70.5 (3)	Li7—O4—Li3—N2	−119.99 (15)
Li6—O6—C6—C7	167.76 (16)	Li7—O4—Li3—C4^i^	122.16 (12)
Li6—O6—C6A—C5A	12 (5)	Li7—O4—Li3—Li6^i^	47.16 (15)
Li6—O6—C6A—C7	152.8 (9)	Li7—O4—Li3—Li4	73.93 (12)
Li6—O6—Li5—O5	−80.33 (14)	Li7—O4—Li3—Li2	−1.80 (12)
Li6—O6—Li5—O3^i^	21.38 (10)	Li7—O2—C19—C20	−157.92 (13)
Li6—O6—Li5—N3	165.34 (16)	Li7—O2—C19—C21	76.63 (15)

Symmetry code: (i) −*x*+3/2, −*y*+1/2, −*z*+1.

### {3-[(2-Methoxyethyl)(methyl)amino]-1,1-dimethylpropanolato}diisopropanolsodium(I) (2) Crystal data

**Table:**

[Na(C_3_H_8_O)_2_(C_8_H_18_NO_2_)]	*Z* = 2
*M**_r_* = 303.41	*F*(000) = 336
Triclinic, *P*1	*D*_x_ = 1.095 Mg m^−^^3^
*a* = 9.9339 (6) Å	Mo *K*α radiation, λ = 0.71073 Å
*b* = 10.3002 (7) Å	Cell parameters from 7656 reflections
*c* = 11.0874 (7) Å	θ = 2.3–27.9°
α = 103.333 (2)°	µ = 0.10 mm^−^^1^
β = 108.132 (2)°	*T* = 100 K
γ = 111.845 (2)°	Block, clear yellowish colourless
*V* = 920.33 (10) Å^3^	0.29 × 0.27 × 0.19 mm

### {3-[(2-Methoxyethyl)(methyl)amino]-1,1-dimethylpropanolato}diisopropanolsodium(I) (2) Data collection

**Table:**

Bruker D8 VENTURE area detector diffractometer	4399 independent reflections
Radiation source: microfocus sealed X-ray tube, Incoatec Iµs	3558 reflections with *I* > 2σ(*I*)
HELIOS mirror optics monochromator	*R*_int_ = 0.040
Detector resolution: 10.4167 pixels mm^-1^	θ_max_ = 27.9°, θ_min_ = 2.3°
ω and φ scans	*h* = −13→13
Absorption correction: multi-scan (SADABS; Bruker, 2016)	*k* = −13→13
*T*_min_ = 0.703, *T*_max_ = 0.746	*l* = −14→14
21708 measured reflections	

### {3-[(2-Methoxyethyl)(methyl)amino]-1,1-dimethylpropanolato}diisopropanolsodium(I) (2) Refinement

**Table:**

Refinement on *F*^2^	Primary atom site location: dual
Least-squares matrix: full	Hydrogen site location: mixed
*R*[*F*^2^ > 2σ(*F*^2^)] = 0.039	H atoms treated by a mixture of independent and constrained refinement
*wR*(*F*^2^) = 0.095	*w* = 1/[σ^2^(*F*_o_^2^) + (0.0375*P*)^2^ + 0.4014*P*] where *P* = (*F*_o_^2^ + 2*F*_c_^2^)/3
*S* = 1.03	(Δ/σ)_max_ < 0.001
4399 reflections	Δρ_max_ = 0.31 e Å^−^^3^
197 parameters	Δρ_min_ = −0.26 e Å^−^^3^
0 restraints	

### {3-[(2-Methoxyethyl)(methyl)amino]-1,1-dimethylpropanolato}diisopropanolsodium(I) (2) Special details

**Table:**

Geometry. All esds (except the esd in the dihedral angle between two l.s. planes) are estimated using the full covariance matrix. The cell esds are taken into account individually in the estimation of esds in distances, angles and torsion angles; correlations between esds in cell parameters are only used when they are defined by crystal symmetry. An approximate (isotropic) treatment of cell esds is used for estimating esds involving l.s. planes.

### {3-[(2-Methoxyethyl)(methyl)amino]-1,1-dimethylpropanolato}diisopropanolsodium(I) (2) Fractional atomic coordinates and isotropic or equivalent isotropic displacement parameters (Å^2^)

**Table:**

	*x*	*y*	*z*	*U*_iso_*/*U*_eq_	
Na1	0.56574 (6)	0.36139 (5)	0.41374 (5)	0.01326 (12)	
O1	0.31637 (10)	0.26510 (9)	0.41304 (8)	0.01364 (18)	
O2	0.70291 (11)	0.22028 (10)	0.38157 (9)	0.0188 (2)	
O3	0.62550 (12)	0.56549 (10)	0.34999 (10)	0.0225 (2)	
H3	0.649 (3)	0.639 (2)	0.430 (2)	0.057 (6)*	
O4	0.77531 (11)	0.54064 (10)	0.63765 (9)	0.0183 (2)	
H4	0.761 (2)	0.617 (2)	0.634 (2)	0.043 (5)*	
N1	0.37916 (13)	0.11246 (11)	0.19792 (10)	0.0154 (2)	
C1	0.23564 (15)	0.10579 (13)	0.35343 (12)	0.0144 (2)	
C2	0.32290 (17)	0.04561 (14)	0.44729 (13)	0.0198 (3)	
H2A	0.4359	0.0885	0.4627	0.030*	
H2B	0.2701	−0.0655	0.4032	0.030*	
H2C	0.3184	0.0754	0.5359	0.030*	
C3	0.06061 (16)	0.04123 (15)	0.33445 (15)	0.0249 (3)	
H3A	0.0601	0.0685	0.4250	0.037*	
H3B	0.0047	−0.0698	0.2870	0.037*	
H3C	0.0052	0.0837	0.2793	0.037*	
C4	0.22490 (15)	0.05000 (14)	0.20708 (13)	0.0172 (3)	
H4A	0.1759	−0.0622	0.1703	0.021*	
H4B	0.1511	0.0760	0.1467	0.021*	
C5	0.35604 (18)	0.14141 (16)	0.07181 (14)	0.0248 (3)	
H5A	0.3224	0.2199	0.0758	0.037*	
H5B	0.2725	0.0475	−0.0083	0.037*	
H5C	0.4577	0.1762	0.0635	0.037*	
C6	0.45368 (16)	0.01318 (14)	0.20398 (13)	0.0191 (3)	
H6A	0.4033	−0.0684	0.1113	0.023*	
H6B	0.4325	−0.0354	0.2681	0.023*	
C7	0.63388 (16)	0.09934 (14)	0.25142 (14)	0.0203 (3)	
H7A	0.6810	0.0305	0.2600	0.024*	
H7B	0.6566	0.1405	0.1835	0.024*	
C8	0.87449 (17)	0.28899 (17)	0.44783 (16)	0.0267 (3)	
H8A	0.9179	0.3183	0.3845	0.040*	
H8B	0.9062	0.2164	0.4740	0.040*	
H8C	0.9174	0.3796	0.5306	0.040*	
C9	0.71232 (16)	0.63586 (15)	0.28242 (13)	0.0213 (3)	
H9	0.8134	0.7301	0.3534	0.026*	
C10	0.75795 (19)	0.52768 (19)	0.20825 (17)	0.0336 (4)	
H10A	0.8185	0.4977	0.2742	0.050*	
H10B	0.8250	0.5785	0.1674	0.050*	
H10C	0.6598	0.4370	0.1353	0.050*	
C11	0.6126 (2)	0.67931 (18)	0.18342 (16)	0.0326 (4)	
H11A	0.5125	0.5879	0.1140	0.049*	
H11B	0.6738	0.7279	0.1377	0.049*	
H11C	0.5866	0.7504	0.2341	0.049*	
C12	0.79279 (16)	0.53749 (14)	0.76930 (13)	0.0197 (3)	
H12	0.7092	0.5563	0.7902	0.024*	
C13	0.95946 (19)	0.65927 (16)	0.88120 (15)	0.0332 (4)	
H13A	1.0419	0.6399	0.8626	0.050*	
H13B	0.9682	0.6570	0.9711	0.050*	
H13C	0.9757	0.7594	0.8820	0.050*	
C14	0.76561 (19)	0.38057 (16)	0.76273 (15)	0.0278 (3)	
H14A	0.6584	0.3038	0.6892	0.042*	
H14B	0.7728	0.3745	0.8513	0.042*	
H14C	0.8481	0.3620	0.7435	0.042*	

### {3-[(2-Methoxyethyl)(methyl)amino]-1,1-dimethylpropanolato}diisopropanolsodium(I) (2) Atomic displacement parameters (Å^2^)

**Table:**

	*U*^11^	*U*^22^	*U*^33^	*U*^12^	*U*^13^	*U*^23^
Na1	0.0173 (2)	0.0096 (2)	0.0137 (2)	0.00671 (19)	0.00710 (19)	0.00498 (18)
O1	0.0187 (4)	0.0083 (4)	0.0151 (4)	0.0065 (3)	0.0084 (3)	0.0050 (3)
O2	0.0185 (4)	0.0172 (4)	0.0207 (5)	0.0102 (4)	0.0087 (4)	0.0047 (4)
O3	0.0383 (6)	0.0133 (4)	0.0160 (5)	0.0088 (4)	0.0148 (4)	0.0080 (4)
O4	0.0246 (5)	0.0152 (4)	0.0149 (4)	0.0122 (4)	0.0052 (4)	0.0058 (3)
N1	0.0219 (5)	0.0136 (5)	0.0129 (5)	0.0095 (4)	0.0089 (4)	0.0054 (4)
C1	0.0178 (6)	0.0092 (5)	0.0158 (6)	0.0057 (5)	0.0080 (5)	0.0046 (4)
C2	0.0325 (7)	0.0136 (6)	0.0188 (6)	0.0129 (5)	0.0130 (6)	0.0094 (5)
C3	0.0210 (7)	0.0195 (6)	0.0297 (7)	0.0055 (5)	0.0137 (6)	0.0062 (6)
C4	0.0180 (6)	0.0146 (6)	0.0135 (6)	0.0060 (5)	0.0046 (5)	0.0028 (5)
C5	0.0336 (8)	0.0288 (7)	0.0165 (6)	0.0158 (6)	0.0133 (6)	0.0117 (6)
C6	0.0276 (7)	0.0116 (6)	0.0197 (6)	0.0104 (5)	0.0126 (5)	0.0041 (5)
C7	0.0280 (7)	0.0167 (6)	0.0232 (7)	0.0139 (6)	0.0161 (6)	0.0073 (5)
C8	0.0203 (7)	0.0272 (7)	0.0339 (8)	0.0129 (6)	0.0118 (6)	0.0115 (6)
C9	0.0227 (7)	0.0175 (6)	0.0164 (6)	0.0036 (5)	0.0073 (5)	0.0068 (5)
C10	0.0298 (8)	0.0431 (9)	0.0314 (8)	0.0205 (7)	0.0144 (7)	0.0137 (7)
C11	0.0522 (10)	0.0304 (8)	0.0235 (7)	0.0231 (8)	0.0178 (7)	0.0168 (6)
C12	0.0245 (7)	0.0172 (6)	0.0144 (6)	0.0112 (5)	0.0041 (5)	0.0056 (5)
C13	0.0373 (9)	0.0204 (7)	0.0215 (7)	0.0098 (6)	−0.0032 (6)	0.0048 (6)
C14	0.0341 (8)	0.0192 (7)	0.0243 (7)	0.0113 (6)	0.0061 (6)	0.0111 (6)

### {3-[(2-Methoxyethyl)(methyl)amino]-1,1-dimethylpropanolato}diisopropanolsodium(I) (2) Geometric parameters (Å, º)

**Table:**

Na1—Na1^i^	3.9154 (9)	C5—H5A	0.9800
Na1—O1	2.2970 (10)	C5—H5B	0.9800
Na1—O2	2.3736 (10)	C5—H5C	0.9800
Na1—O3	2.2998 (10)	C6—H6A	0.9900
Na1—H3	2.61 (2)	C6—H6B	0.9900
Na1—O4	2.3905 (10)	C6—C7	1.5116 (19)
Na1—H4	2.637 (19)	C7—H7A	0.9900
Na1—N1	2.5707 (11)	C7—H7B	0.9900
O1—C1	1.4012 (13)	C8—H8A	0.9800
O2—C7	1.4225 (15)	C8—H8B	0.9800
O2—C8	1.4244 (16)	C8—H8C	0.9800
O3—H3	0.92 (2)	C9—H9	1.0000
O3—C9	1.4205 (16)	C9—C10	1.519 (2)
O4—H4	0.86 (2)	C9—C11	1.505 (2)
O4—C12	1.4249 (15)	C10—H10A	0.9800
N1—C4	1.4717 (16)	C10—H10B	0.9800
N1—C5	1.4629 (16)	C10—H10C	0.9800
N1—C6	1.4705 (15)	C11—H11A	0.9800
C1—C2	1.5345 (17)	C11—H11B	0.9800
C1—C3	1.5353 (18)	C11—H11C	0.9800
C1—C4	1.5463 (17)	C12—H12	1.0000
C2—H2A	0.9800	C12—C13	1.5206 (19)
C2—H2B	0.9800	C12—C14	1.5163 (18)
C2—H2C	0.9800	C13—H13A	0.9800
C3—H3A	0.9800	C13—H13B	0.9800
C3—H3B	0.9800	C13—H13C	0.9800
C3—H3C	0.9800	C14—H14A	0.9800
C4—H4A	0.9900	C14—H14B	0.9800
C4—H4B	0.9900	C14—H14C	0.9800
			
Na1^i^—Na1—H3	53.0 (5)	N1—C4—H4B	108.4
Na1^i^—Na1—H4	54.7 (4)	C1—C4—H4A	108.4
O1—Na1—Na1^i^	60.32 (2)	C1—C4—H4B	108.4
O1—Na1—O2	124.96 (4)	H4A—C4—H4B	107.4
O1—Na1—O3	113.36 (4)	N1—C5—H5A	109.5
O1—Na1—H3	107.0 (5)	N1—C5—H5B	109.5
O1—Na1—O4	111.89 (4)	N1—C5—H5C	109.5
O1—Na1—H4	107.6 (4)	H5A—C5—H5B	109.5
O1—Na1—N1	73.22 (3)	H5A—C5—H5C	109.5
O2—Na1—Na1^i^	162.16 (3)	H5B—C5—H5C	109.5
O2—Na1—H3	127.7 (5)	N1—C6—H6A	109.2
O2—Na1—O4	93.84 (4)	N1—C6—H6B	109.2
O2—Na1—H4	110.0 (4)	N1—C6—C7	112.24 (10)
O2—Na1—N1	69.39 (3)	H6A—C6—H6B	107.9
O3—Na1—Na1^i^	69.43 (3)	C7—C6—H6A	109.2
O3—Na1—O2	116.52 (4)	C7—C6—H6B	109.2
O3—Na1—H3	20.3 (5)	O2—C7—C6	108.25 (10)
O3—Na1—O4	85.59 (4)	O2—C7—H7A	110.0
O3—Na1—H4	69.9 (4)	O2—C7—H7B	110.0
O3—Na1—N1	109.84 (4)	C6—C7—H7A	110.0
H3—Na1—H4	51.9 (7)	C6—C7—H7B	110.0
O4—Na1—Na1^i^	69.31 (3)	H7A—C7—H7B	108.4
O4—Na1—H3	69.5 (5)	O2—C8—H8A	109.5
O4—Na1—H4	18.9 (4)	O2—C8—H8B	109.5
O4—Na1—N1	160.83 (4)	O2—C8—H8C	109.5
N1—Na1—Na1^i^	126.01 (3)	H8A—C8—H8B	109.5
N1—Na1—H3	128.0 (5)	H8A—C8—H8C	109.5
N1—Na1—H4	179.1 (4)	H8B—C8—H8C	109.5
C1—O1—Na1	110.84 (7)	O3—C9—H9	108.7
C7—O2—Na1	118.31 (7)	O3—C9—C10	109.09 (11)
C7—O2—C8	112.37 (10)	O3—C9—C11	110.25 (12)
C8—O2—Na1	121.71 (8)	C10—C9—H9	108.7
Na1—O3—H3	99.4 (13)	C11—C9—H9	108.7
C9—O3—Na1	141.03 (8)	C11—C9—C10	111.20 (12)
C9—O3—H3	108.7 (13)	C9—C10—H10A	109.5
Na1—O4—H4	96.9 (13)	C9—C10—H10B	109.5
C12—O4—Na1	129.10 (8)	C9—C10—H10C	109.5
C12—O4—H4	107.1 (13)	H10A—C10—H10B	109.5
C4—N1—Na1	104.76 (7)	H10A—C10—H10C	109.5
C5—N1—Na1	111.10 (8)	H10B—C10—H10C	109.5
C5—N1—C4	110.12 (10)	C9—C11—H11A	109.5
C5—N1—C6	110.29 (10)	C9—C11—H11B	109.5
C6—N1—Na1	107.80 (7)	C9—C11—H11C	109.5
C6—N1—C4	112.64 (10)	H11A—C11—H11B	109.5
O1—C1—C2	109.45 (10)	H11A—C11—H11C	109.5
O1—C1—C3	111.07 (10)	H11B—C11—H11C	109.5
O1—C1—C4	109.85 (9)	O4—C12—H12	109.1
C2—C1—C3	108.59 (10)	O4—C12—C13	110.66 (12)
C2—C1—C4	111.93 (10)	O4—C12—C14	108.08 (11)
C3—C1—C4	105.91 (10)	C13—C12—H12	109.1
C1—C2—H2A	109.5	C14—C12—H12	109.1
C1—C2—H2B	109.5	C14—C12—C13	110.77 (11)
C1—C2—H2C	109.5	C12—C13—H13A	109.5
H2A—C2—H2B	109.5	C12—C13—H13B	109.5
H2A—C2—H2C	109.5	C12—C13—H13C	109.5
H2B—C2—H2C	109.5	H13A—C13—H13B	109.5
C1—C3—H3A	109.5	H13A—C13—H13C	109.5
C1—C3—H3B	109.5	H13B—C13—H13C	109.5
C1—C3—H3C	109.5	C12—C14—H14A	109.5
H3A—C3—H3B	109.5	C12—C14—H14B	109.5
H3A—C3—H3C	109.5	C12—C14—H14C	109.5
H3B—C3—H3C	109.5	H14A—C14—H14B	109.5
N1—C4—C1	115.58 (10)	H14A—C14—H14C	109.5
N1—C4—H4A	108.4	H14B—C14—H14C	109.5
			
Na1—O1—C1—C2	67.90 (10)	O1—C1—C4—N1	53.41 (13)
Na1—O1—C1—C3	−172.20 (8)	N1—C6—C7—O2	56.12 (13)
Na1—O1—C1—C4	−55.36 (10)	C2—C1—C4—N1	−68.39 (13)
Na1—O2—C7—C6	−41.77 (12)	C3—C1—C4—N1	173.44 (10)
Na1—O3—C9—C10	13.35 (19)	C4—N1—C6—C7	−157.08 (10)
Na1—O3—C9—C11	135.73 (12)	C5—N1—C4—C1	−142.14 (11)
Na1—O4—C12—C13	169.52 (8)	C5—N1—C6—C7	79.44 (13)
Na1—O4—C12—C14	48.07 (14)	C6—N1—C4—C1	94.29 (12)
Na1—N1—C4—C1	−22.60 (11)	C8—O2—C7—C6	168.09 (11)
Na1—N1—C6—C7	−42.01 (11)		

Symmetry code: (i) −*x*+1, −*y*+1, −*z*+1.

### {3-[(2-Methoxyethyl)(methyl)amino]-1,1-dimethylpropanolato}diisopropanolsodium(I) (2) Hydrogen-bond geometry (Å, º)

**Table:**

*D*—H···*A*	*D*—H	H···*A*	*D*···*A*	*D*—H···*A*
O3—H3···O1^i^	0.92 (2)	1.65 (2)	2.5442 (12)	165 (2)
O4—H4···O1^i^	0.86 (2)	1.75 (2)	2.5894 (12)	164.4 (19)

Symmetry code: (i) −*x*+1, −*y*+1, −*z*+1.
